# Uncovering pseudotemporal trajectories with covariates from single cell and bulk expression data

**DOI:** 10.1038/s41467-018-04696-6

**Published:** 2018-06-22

**Authors:** Kieran R Campbell, Christopher Yau

**Affiliations:** 1 Department of Physiology, Anatomy and Genetics, University of Oxford, Oxford, OX1 3QX UK; 2 Wellcome Trust Centre for Human Genetics, University of Oxford, Oxford, OX3 7BN UK; 3 Centre for Computational Biology, Institute of Cancer and Genomic Sciences, University of Birmingham, Birmingham, B15 2TT UK; 4 Present Address: Department of Statistics, University of British Columbia, Vancouver, BC V6T 1Z4 Canada

## Abstract

Pseudotime algorithms can be employed to extract latent temporal information from cross-sectional data sets allowing dynamic biological processes to be studied in situations where the collection of time series data is challenging or prohibitive. Computational techniques have arisen from single-cell ‘omics and cancer modelling where pseudotime can be used to learn about cellular differentiation or tumour progression. However, methods to date typically implicitly assume homogeneous genetic, phenotypic or environmental backgrounds, which becomes limiting as data sets grow in size and complexity. We describe a novel statistical framework that learns how pseudotime trajectories can be modulated through covariates that encode such factors. We apply this model to both single-cell and bulk gene expression data sets and show that the approach can recover known and novel covariate-pseudotime interaction effects. This hybrid regression-latent variable model framework extends pseudotemporal modelling from its most prevalent area of single cell genomics to wider applications.

## Abstract (web summary)

Cross-sectional omic data often have non-homogeneous genetic, phenotypic, or environmental backgrounds. Here, the authors develop a statistical framework to infer pseudotime trajectories in the presence of such factors as well as their interactions in both single-cell and bulk gene expression analysis

## Introduction

Dynamic or progressive biological behaviour are ideally studied within a longitudinal framework that allows for monitoring of individuals over time leading to direct time course data. However, longitudinal studies are often challenging to conduct and cohort sizes limited by logistical and resource availability. In contrast, cross-sectional surveys of a population are relatively easier to conduct in large numbers and more prevalent for molecular ‘omics based studies. Cross-sectional studies do not directly capture the changes in disease characteristics in patients but it may be possible to recapitulate aspects of temporal variation by applying “pseudotime” computational analysis.

**Fig. 1:**
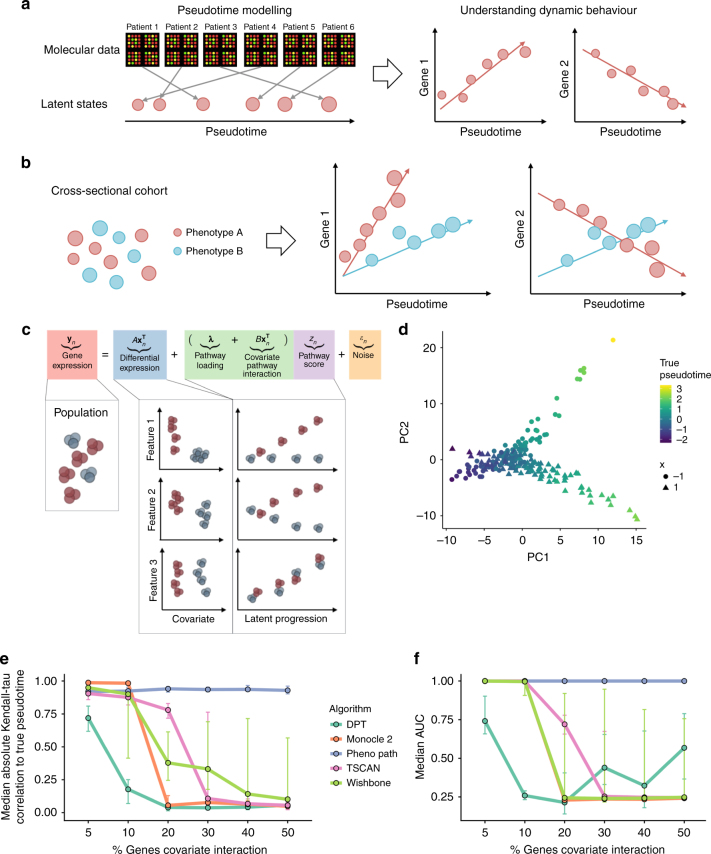
An illustration of pseudotemporal analysis. **a** High-dimensional molecular data from a cross-sectional cohort is mapped on to a one-dimensional pseudotemporal progression scale allowing pseudotemporal behaviour of individual features to be analysed. **b** If the cohort contains sub-populations we may want each sub-population to be associated to distinct trajectories. **c** PhenoPath models observed expression as a combination of standard differential expression and pseudotime/pathway effects, including covariate-pathway interactions. **d** PCA representation of a simulated data set coloured by pseudotime shows a clear splitting of trajectories between covariate status *x* = (−1, 1). **e** Median Kendall-*τ* correlation to true pseudotime across varying fractions of genes exhibiting covariate-trajectory interactions on simulated data. PhenoPath is the only algorithm for which the accuracy of inference is independent of the (unknown) fraction. **f** Median AUCs measuring the accuracy of different approaches to detecting covariate-trajectory interactions using Limma Voom for differential expression analysis. As before, PhenoPath is the only algorithm for which the accuracy is independent of the underlying fraction of genes exhibiting covariate-trajectory interactions

The objective of pseudotime analysis is to take a collection of high-dimensional molecular data from a cross-sectional cohort of individuals and to map these on to a series of one-dimensional quantities, called *pseudotimes*. These pseudotimes measure the relative progression of each of the individuals along the biological process of interest, e.g., disease progression, cellular development, etc., allowing us to understand the (pseudo)temporal behaviour of measured features without explicit time series data (Fig. 1a). This analysis is possible when individuals in the cross-sectional cohort behave asynchronously and each is at a different stage of progression. Therefore, by creating a relative ordering of the individuals, we can define a series of molecular states that constitute a trajectory for the process of interest.

Pseudotime methods generally rely on the assumption that any two individuals with similar observations should carry correspondingly similar pseudotimes and algorithms will attempt to find some ordering of the individuals that satisfies some overall global measure that best adheres to this assumption (Fig. 1a). Exact implementations and specifications differ between pseudotime approaches particularly in the way “similarity” is defined. When applied to molecular data, pseudotime analysis typically captures some dominant mode of variation that corresponds to the continuous (de)activation of a set of biological pathways^1^.

Pseudotime analysis has gained particular popularity in the domain of single-cell gene expression analysis (where each “individual” is now a single cell) in which it has been applied to model the differentiation of single-cells^2–9^ (a comprehensive catalogue of single-cell pseudotime algorithms can be obtained from https://github.com/agitter/single-cell-pseudotime). Using advanced machine learning techniques, these methods can be applied to characterise complex, nonlinear behaviours, such as cell cycle, and modelling branching behaviours to allow, for example, the possibility of cell fate decision making and lineage reconstruction. However, these single-cell applications were pre-dated by more general applications in modeling cancer progression^10–12^, as well as other progressive diseases^13–16^. Examples of such work provided early inspiration for single-cell pseudotime methods, e.g., Monocle^2^. To date, there has been little cross-over between these distinct application domains in terms of methodological development due to the different contexts in which methods are applied. However, there are interesting possibilities that could arise by translating recent advances in single-cell pseudotime modelling and applying these to tackling related problems in disease progression modelling. This is the topic of the work presented here.

We focus on a variant of pseudotime analysis that has previously been unexplored. While recent single-cell pseudotime approaches provide powerful means for unsupervised identification of single or multiple, branching pseudotime trajectories, these can only be retrospectively examined for their association with prior factors of interest. We sought to develop a statistical model in which these factors could be explicitly incorporated into pseudotime analysis. This capability is important as it would provide a mechanism to account for known genetic, phenotypic or environmental factors allowing gene expression variability to be decomposed into different contributory factors. Doing so would allow us to answer questions related to the interaction between heterogeneity in these external factors and biological progression. For example, how does cellular development differ when cells are exposed to different stimuli? Does the evolution of transcriptional programming in cancer depend on the histopathological classification of the tumours?

We describe a novel Bayesian statistical framework for pseudotime trajectory modelling that allows explicit inclusion of prior factors of interest. Our approach allows us to incorporate information in the form of covariates that can modulate the pseudotemporal progression allowing sub-groups within the cross-sectional population to each develop their own trajectory (Fig. 1b). Our approach combines linear regression and latent variable modelling and allows for interactions between the covariates and temporally driven components of the model. We believe our method to be the first integrated statistical approach to allow for modelling pseudotime trajectories on heterogeneous backgrounds allowing its utility in both single and non-single cell applications.

## Results

### A Bayesian approach for pseudotemporal learning with covariates

We first give an overview of our statistical method which we call “PhenoPath”. For simplicity, our descriptions will assume that the observed data are high-dimensional gene expression measurements which are used throughout our empirical experiments but we stress that the model would be applicable to a wider range of data modalities.

The objective of PhenoPath is to provide a probabilistic ordering of high-dimensional gene expression measurements across objects (e.g., cells, tumours, patients, etc) (see Fig. 1a). This is achieved by compressing the information contained within the data on to a unidimensional axis. Our aim is to construct an axis such that relative positions along the axis correspond to some meaningful biological or disease progression. The novelty of PhenoPath is to introduce the notion that our objects may have different labels (covariates) attached to them corresponding to different innate properties or exposure to external stimuli. These factors might cause the objects to evolve over (pseudo)time differently (Fig. 1b). The result is that PhenoPath simultaneously learns a pseudotemporal axis that is common to the different object labels, while decomposing gene expression variability into static and dynamic components.

More specifically, PhenoPath uses a Bayesian statistical framework that integrates linear regression and latent variable modelling. The observed data (**y**_*n*_) for the *n*th individual is a linear function of both measured covariates (**x**_*n*_) and an unobserved latent variable (*z*_*n*_) corresponding to latent progression that we will term pseudotime.

A schematic relating the parameters in the overall model is shown in Fig. 1c. In PhenoPath, the model involves three components: (i) gene expression is determined by a static component based on your covariate status (${A}{\mathbf{x}}_n^{\mathrm{T}}$), (ii) a dynamic component related to how far along the biological process you are (***λ****z*_*n*_) and the main novelty (iii) an interaction component which allows your covariate status to change the direction of the dynamic component of the gene expression (${B}{\mathbf{x}}_n^{\mathrm{T}}z_n$). PhenoPath reduces down to linear regression based differential expression analysis or factor analysis based pseudotime analysis if only the first or second components are used respectively. Standard models are therefore nested within PhenoPath.

In our investigations, the covariates will be binary quantities but this is not a necessary restriction and in practice any arbitrary design matrix that can be used for standard regression may be used for **x** (Supplementary Results). Sparse Bayesian prior probability distributions are used to constrain the parameters (*A*, *B*, ***λ***) so that covariates only drive the emergence of distinct trajectories if there is sufficient information within the data to do so. Computational inference within PhenoPath is handled by a fast and highly scalable variational Bayesian inference framework that can handle thousands of features and samples in minutes using a standard personal computer making it readily applicable to large data sets without the use of high-performance computing (see Methods section for details). Though variational inference of such hierarchical Bayesian models can be sensitive to hyperparameters values and parameter initialisation we found PhenoPath to be robust to such choices by fitting on over 80 combinations of (hyper)parameter initialisation (Supplementary Results).

### Simulation study

We first developed a simulation study to assess the performance of our model relative to existing approaches for pseudotime estimation and differential expression analyses for situations in which pseudotime trajectories are modulated by covariate status. To do this we simulated pseudotemporally regulated RNA-seq data from a nonlinear mean function with a negative binomial noise distribution. This is an entirely different generative process to that assumed by PhenoPath and designed to test for robustness to model misspecification. We generated simulated data sets containing gene sets involving 5, 10, 20, …, 50% of genes with covariate-trajectory interactions. An example is shown in Fig. 1d where the direction of the pseudotime trajectory depends on whether the artificial covariate *x* = −1 or *x* = 1. This was repeated for data sets involving 200 and 500 samples, for high and low noise regimes with 40 replicates per condition, giving 960 distinct data sets (see Supplementary Methods and Results).

We applied PhenoPath and four state-of-the-art pseudotime algorithms: Monocle 2^8^, Diffusion Pseudotime (DPT^5^), Wishbone^7^ and TSCAN^3^. We measured the median Kendall-*τ* correlation between the inferred pseudotimes and the true pseudotimes used in the simulations (Fig. 1e). Our results showed that when the fraction of genes exhibiting covariate-trajectory interactions is small (5%), all approaches perform well. However, as expected, as this fraction increased (>10%), the performance of PhenoPath remains consistent while the others diminished rapidly since the latter do not account for such interaction effects.

Next, for each data set and each pseudotime analysis, we performed differential expression analysis testing for covariate-trajectory interactions using Limma Voom^17^, DESeq2^18^, MAST^19^ and Monocle 2^8^. This gave a total of 35,520 distinct differential expression workflows (full details in Supplementary Results). The accuracy of each method to identify interactions was assessed using the area under the receiver-operator curve (AUC). Again, when the fraction of genes exhibiting covariate-trajectory interactions is small (5–10%) then all algorithms perform well at identifying interactions with high AUCs (Fig. 1f). However, as this fraction increases, the AUC of all algorithms other than PhenoPath rapidly decreases, while PhenoPath maintains the ability to detect interactions.

Overall, our simulations showed that if pseudotime trajectories are modulated by covariate status, then the application of standard pseudotime algorithms may be sub-optimal if there are a number of such interactions. For real data sets, where the underlying fraction of covariate-trajectory interactions would be unknown a priori, the uniformity of PhenoPath performance in these simulations is advantageous. Furthermore, our integrated model is more powerful than a two-stage procedure in which pseudotime is fitted first and then standard differential analysis applied since if pseudotime is incorrectly estimated at the first stage, covariate-trajectory interactions will not be identified correctly at the second stage (Supplementary Results).

An alternative analysis strategy is to fit pseudotime to subsets of the data—one subset for each covariate value. This approach would only be applicable for discrete covariates where there are sufficient numbers of samples per covariate level but not continuous covariates (PhenoPath can also use continuous covariates). However, pseudotimes would have to be fitted to every combination of the factor levels, resulting in an exponentially increasing number of groups for pseudotime inference and downstream analysis. Furthermore, while this could enable accurate pseudotime estimation for each covariate group, it would be necessary to align the pseudotime trajectories between the groups leading to further algorithmic design and implementation choices. PhenoPath circumvents all of these issues by providing an integrated model for deriving a single universal pseudotime trajectory which is locally modulated for features that vary by covariate status alleviating the requirement to align multiple trajectories. Further discussion of this strategy is explored in Supplementary Results.

### Single-cell RNA-seq perturbation analysis

We next examined a time-series single-cell RNA-seq (scRNA-seq) data set of bone marrow derived dendritic cells responding to particular stimuli^20^. Cells were exposed to LPS, a component of Gram-negative bacteria, and PAM, a synthetic mimic of bacterial lipopeptides, and scRNA-seq performed at 1, 2, 4 and 6 h after stimulation. Using the capture time information, the original study was able to study single-cell gene expression dynamics under the two exposures. However, although capture times were measured, previous analyses have suggested this data set is more suited to a “pseudotime” analysis as the cells respond asynchronously and heterogeneity exists within the cellular populations at each time point^4^.

We conducted an analysis using PhenoPath where we encoded the stimulant to which the cells were exposed as a binary covariate. Each gene was therefore modelled as a combination of static effects due to LPS/PAM exposure, dynamic effects due to temporal variation (independent of stimulant type) and dynamic effects that were modulated by the stimulant. We applied PhenoPath to 820 cells using the 7500 most variable genes from a recent re-quantification of the original data set^21^ using Salmon^22^. The capture times were not used for PhenoPath analysis (details of quality control and data filtering are given in Supplementary Methods).

**Fig. 2:**
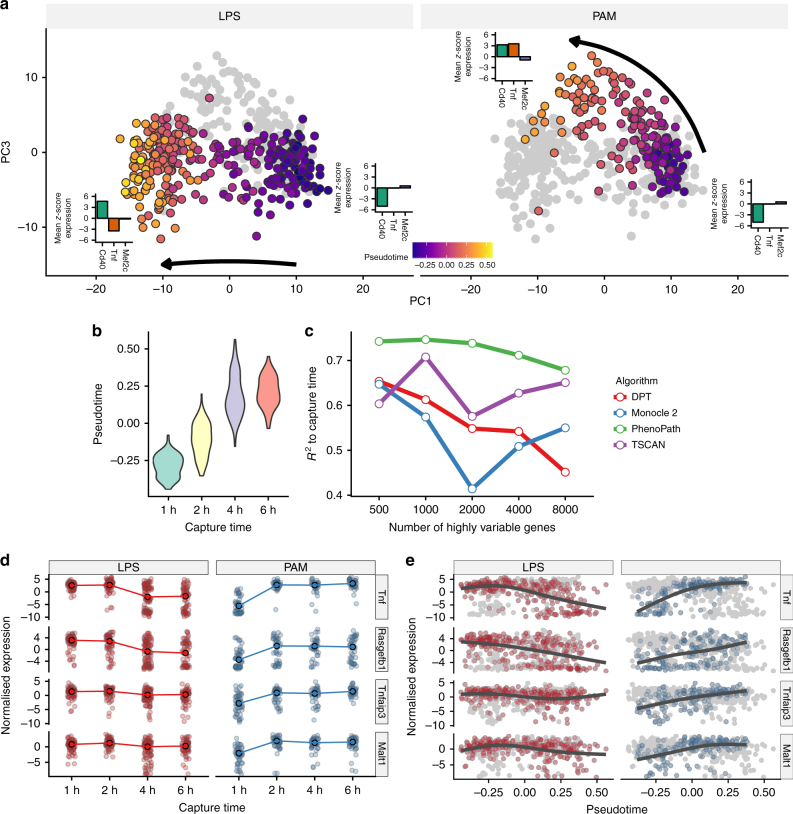
PhenoPath pseudotime analysis of single-cell RNA-sequencing data under perturbation. **a** PCA representation of the PhenoPath pseudotime fit shows distinct trajectories of the dendritic cells under LPS or PAM stimulation, with an initial common cell state before transcriptomic divergence as the different stimulants are applied. **b** The inferred pseudotimes recapitulate the physical progression of the cells with *R*^2^ = 0.68 to the capture times. **c** The pseudotimes inferred by PhenoPath recapitulate the physical progression of the cells more accurately than three state-of-the-art pseudotime algorithms across a range of input genes. **d** Expression of *Tnf*, *Rasgefb1*, *Tnfaip3*, and *Malt1*—the four genes with the largest interaction effects—as a function of physical capture time stratified by applied stimulant. **e** The same four genes from (**d**) as a function of physical capture time. Strikingly, *Tnf* is upregulated under PAM exposure yet downregulated under LPS stimulation

A principal components analysis (PCA) representation of the PhenoPath pseudotime fit is shown in Fig. 2a. Distinct response trajectories of the dendritic cells under either LPS or PAM stimulation are evident, with a common cell state at the beginning of pseudotime diverging under LPS and PAM stimulation. Despite capture times not being used as an input, the PhenoPath pseudotime trajectory recapitulates the physical time progression of the cells with an *R*^2^ = 0.68 (Fig. 2b) with 7500 highly variable genes as input. We compared the ability of PhenoPath to recapitulate the physical progression of the cells through pseudotime inference to three state-of-the-art pseudotime algorithms (Monocle 2^8^, DPT^5^ and TSCAN^3^) across a wide range of gene set sizes. We found that for every input gene set size PhenoPath reported a higher correlation with capture time (Fig. 2c) than other methods tested. Figure 2d depicts the gene expression behaviour for four selected genes based on the original physical capture times that display apparent time-dependent behaviour that depends also on the stimulation applied. PhenoPath trajectories enhance our ability to resolve these trends by aligning the cells under LPS and PAM on to a common pseudotemporal scale without the need to compute separate trajectories (Fig. 2e).

**Fig. 3:**
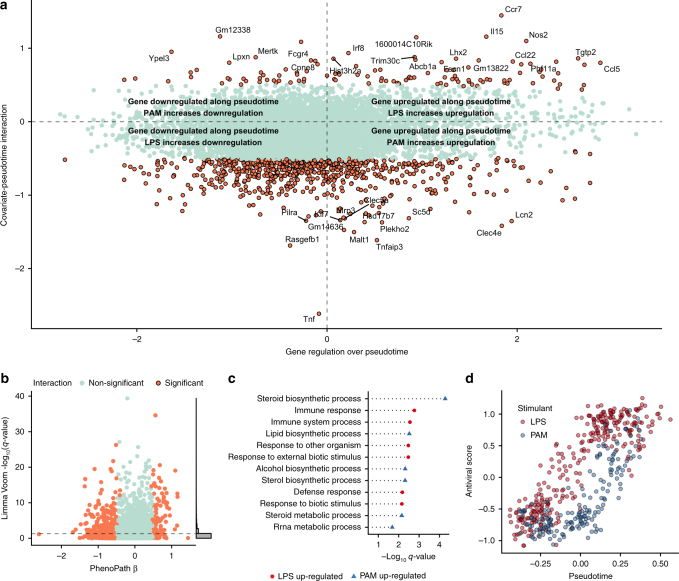
PhenoPath identifies genes differentially modulated over pseudotime using single-cell RNA-sequencing data under perturbation. **a** PhenoPath applied to the Shalek et al. data set uncovers a landscape of genes differentially regulated along pseudotime depending on the stimulant (LPS or PAM) applied. **b** A comparison of *p*-values obtained through a statistical test for differential expression between LPS and PAM stimulation shows no particular relation with the interaction parameters *β* inferred with PhenoPath. **c** A GO enrichment analysis of the genes upregulated along pseudotime whose upregulation was increased by LPS stimulation showed enrichment for immune system processes. **d** Relationship between PhenoPath pseudotime and antiviral score^20^

We next examined the genes whose behaviour over pseudotime were most perturbed by LPS or PAM stimulation. We uncovered a landscape of interactions where the (pseudo)-temporal behaviour of expressed genes depended on whether the cells were exposed to LPS or PAM (Fig. 3a). Figure 2e illustrates four such genes. Most notably, the tumour necrosis factor *Tnf* had around twice the interaction effect size of any other gene, and its expression decreases under LPS stimulation but increases under PAM. Further genes exhibit differential regulation according to stimulant, such as *Mef2c* that has constant expression over pseudotime under LPS stimulation yet shows downregulation under PAM stimulation.

To find out whether these interacting genes would have been identified using a simple differential expression (DE) analysis, we used Limma Voom^17^ to test for stimulant-dependent differences in expression and compared this to the interaction coefficients (*β*) inferred using PhenoPath (Fig. 3b). We found that while some genes that exhibit stimulant-pseudotime interactions can be identified as differentially expressed genes, the majority require the explicit PhenoPath model to resolve the relative contributions of the static and dynamic expression components.

To investigate which biological pathways are perturbed as the cells progress under the different stimulants we performed a Gene Ontology enrichment analysis^23^. Genes whose upregulation was increased over (pseudo-)time by LPS exposure were highly enriched for immune response (Fig. 3c), consistent with previous results^4,20^ that suggest a “core” module of antiviral genes upregulated at later timepoints in LPS cells, though discovered through an entirely unsupervised and integrative methodology. We confirmed this by comparing the inferred pseudotimes of the cells to the antiviral score based on the *Id* gene set from the original publication, finding a strong relationship for both cells stimulated under LPS and PAM (Fig. 3d). Furthermore, of the top 30 significant interactions with negative *β* values (indicating stronger downregulation under LPS) 40% were present in the *IIIc* peaked inflammatory module identified in the original publication, including *Tnf* and *Malt1*. In our analysis, PhenoPath was able to successfully recapitulate previous results (obtained through clustering and manual annotation) in an unsupervised manner without knowledge of the capture times.

### Pseudotemporal modelling in colorectal cancer

We next applied our model in a non-single-cell setting by examining RNA sequencing gene expression data from the TCGA colorectal adenocarcinoma (COAD) cohort^24^ using microsatellite instability (MSI) status as a phenotypic covariate. MSI is genetic hypermutability that is present in ~10–15% of colorectal tumours and is associated with differential response to chemotherapeutics and marginally improved prognosis^25^. Pseudotime inference using PhenoPath was applied to 4801 highly variable genes across 284 COAD samples (details of quality control and data filtering are given in Supplementary Methods).

**Fig. 4:**
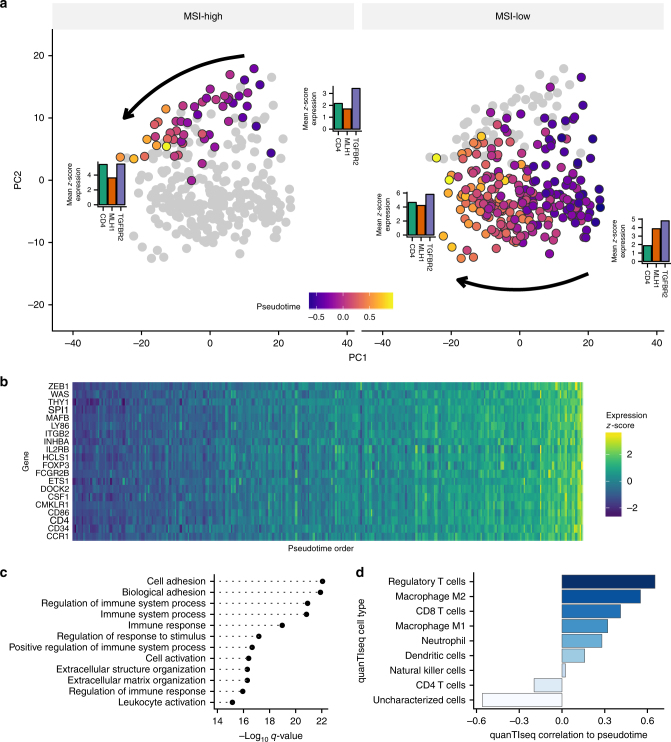
PhenoPath analysis of colorectal adenocarcinoma progression. **a** PCA representation of the trajectory inferred by PhenoPath stratified by microsatellite instability status. **b** Heatmap of expression of immune response genes shows upregulation over pseudotime. **c** A GO enrichment analysis of upregulated genes confirms the latent trajectory encodes immune pathway activation in each tumour. **d** Correlation of quanTIseq tumour immune content to PhenoPath pseudotime

Using PhenoPath we identified a common pseudotemporal scale but distinct development trajectories for MSI-high and MSI-low tumours (Fig. 4a). We observed that the expression of T-regulatory cell (Tregs) immune markers (Fig. 4b) was increased along the trajectory and found, in a Gene Ontology (GO) analysis, an enrichment of immune-related pathways (Fig. 4c). This suggested that PhenoPath has ordered the tumours according to levels of tumour immunogenicity and Tregs infiltration of the tumours. This is consistent with Tregs acting as potent immunosuppressive cells of the immune system and promote progression of cancer through their ability to limit anti-tumour immunity^26^. To corroborate this proposition, we used an bulk RNA sequencing deconvolution tool, quanTIseq^27^, which uses transcriptomic profiles of immune cells to estimate immune cell content of each tumour (Fig. 4d). We found that tumours identified by quanTIseq as having high regulatory T cell or immune cell content scores were most correlated with PhenoPath pseudotime implying that PhenoPath had unbiasedly identified an immunogenic contribution to colorectal cancer progression through unsupervised analysis.

**Fig. 5:**
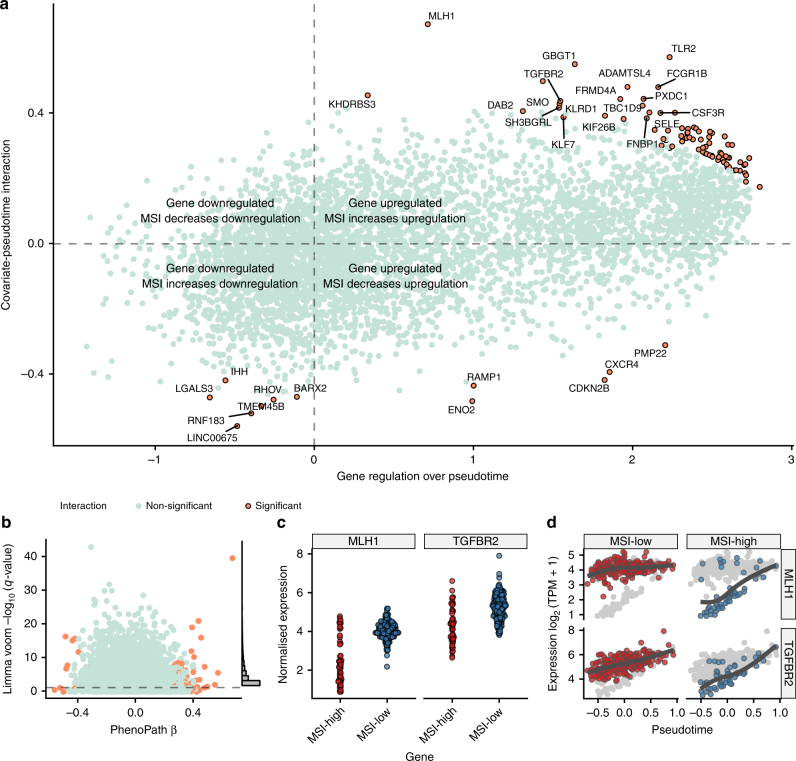
Immune-microsatellite instability interactions in colorectal adenocarcinoma. **a** PhenoPath applied to colorectal adenocarcinoma (COAD) RNA-seq expression data uncovers a landscape of interactions between the inferred immune trajectory and microsatellite instability status (MSI). **b** A comparison to the FDR-corrected *q*-values reported by Limma Voom demonstrates genes found interacting with MSI status and the immune pathway are found to be both DE and non-DE in standard analyses. **c** Standard differential expression analysis masks the relationship between immune response and microsatellite instability in the expression of *MLH1* and *TGFBR2*. **d** Using PhenoPath pseudotime the expression of *MLH1* and *TGFBR2* were identified as being significantly perturbed along the immune trajectory by MSI status. *MLH1* shows no interaction with immune pathway activation in the MSI-low regime yet is highly correlated with immune pathway activation in the MSI-high regime

We next examined 92 putative covariate-pseudotime interactions including known tumour suppressor genes (Fig. 5a). Importantly, PhenoPath identified the *MLH1* gene whose interaction effect size was far larger than any other gene. This association provides an important positive control since *MLH1* is a well-known DNA mismatch repair gene. Germline mutations in *MLH1* are causal for hereditary non-polyposis colorectal cancer^28,29^ while epigenetic silencing in sporadic CRCs is associated with MSI.

We performed a standard differential expression analysis to determine differences between MSI groups using limma voom^17^ (Fig. 5b). Whilst many of these 92 genes are differentially expressed between MSI groups, including *MLH2* and *TGFBR2* (Fig. 5c), PhenoPath is able to resolve the dynamic contribution to these expression differences (Fig. 5d). In this case, while the expression of these genes in MSI-low tumours is relatively constant, in MSI-high tumours, there is a spectrum of expression levels that linearly changes over pseudotime following the increasing immune cell infiltration in the MSI-high tumours.

We next sought to uncover whether the other genes exhibiting interactions between the immune response and microsatellite instability displayed a concerted action in any cancer-related pathways. We took the top 20 genes by interaction effect size and performed an unsupervised pathway enrichment analysis using Reactome^30^. At an FDR <5% we found these genes were enriched for *RUNX1/RUNX2* regulates genes involved in differentiation of myeloid cells. This enrichment was due to the presence of the gene *LGALS3*^31^ that was found to exhibit interactions by PhenoPath. The protein Galactin-3 is encoded by *LGALS3* and altered expression of galectins in human gastrointestinal tissues as being implicated in colorectal cancer progression^32^.

### Pseudotemporal modelling in breast cancer

We finally performed a pseudotemporal analysis of the TCGA breast cancer cohort using estrogen receptor (ER) status as a phenotypic covariate. Approximately 60% of breast cancers are estrogen receptor positive^33^, which is typically associated with improved prognosis and a longer time to recurrence^34^.

**Fig. 6:**
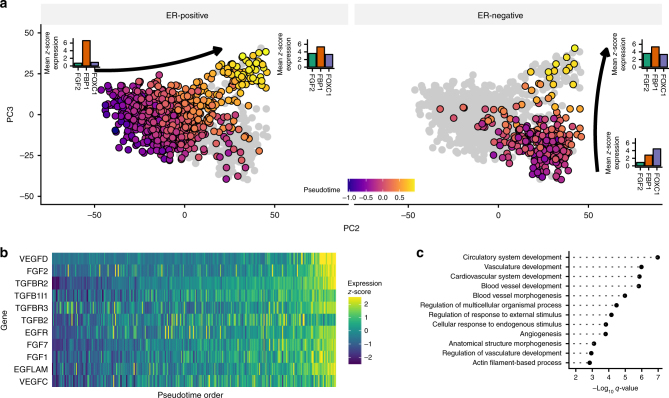
Vascular growth trajectories uncovered by PhenoPath in breast cancer. **a** Principal components visualisation of ER− dependent pseudotemporal trajectories. **b** Expression of a number of vascular growth markers shows upregulation over the inferred pseudotime trajectory. **c** A GO enrichment analysis of upregulated genes confirms the latent trajectory encodes angiogenesis pathway activation in each tumour

We applied PhenoPath to 1135 breast cancers over 4579 highly variable genes and identified distinct ER status specific pseudotemporal trajectories (Fig. 6a). Details of quality control and data filtering are given in Supplementary Methods. We found that markers of vascular growth pathways or angiogenesis—a well-known and uncontroversial hallmark of cancer development^35,36^ - showed common pseudotemporal progression independent of ER status. This included fibroblast growth factor-2 (*FGF2*) and vascular endothelial growth factors C and D (*VEGFC/VEGFD*) (Fig. 6b). A GO enrichment analysis indicated that the genes driving the inferred pseudotemporal trajectory were indeed enriched for vascular growth pathways (Fig. 6c). Through unsupervised analysis, PhenoPath had ordered the breast tumours and measured breast tumour progression in terms of angiogenic development. Survival analysis using stratified (by ER status) Cox proportional hazards modelling with covariates suggested that the pseudotime covariate was significant (*p* = 0.0032). This gave evidence that increasing pseudotemporal progression in these breast tumours conferred reduced overall survival rates (Supplementary Results; Supplementary Fig. 17).

**Fig. 7:**
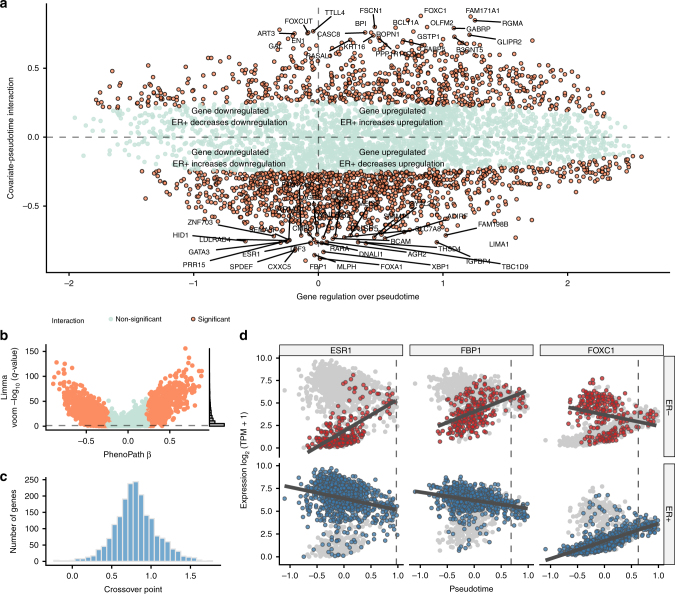
Vascular growth-ER status interactions uncovered by PhenoPath in breast cancer. **a** PhenoPath applied to Breast Cancer (BRCA) RNA-seq expression data uncovers a landscape of interactions between the inferred angiogenesis trajectory and estrogen receptor (ER) status. **b** A comparison to the FDR-corrected *q*-values reported by Limma Voom identifies a significant number of DE genes display an interaction with ER status and the anthropogenic pathway. **c** A histogram of the cross-over points of all genes whose trajectory-covariate interactions were significant. The vast majority of cross-over points are at the end of the trajectory (around 0.8, where the “middle” pathway score is 0) implying a convergence of gene expression as the trajectory progresses. **d** Three example genes *ESR1*, *FBP1*, and (*FOXC1*) were identified by PhenoPath as significantly perturbed along the angiogenesis trajectory by ER status. The vertical dashed line signifies the calculated cross-over point, demonstrating the expression profiles of these genes converge towards the end of the trajectory

In order to understand how angiogenic development differs by ER status, we examined the landscape of genes exhibiting covariate-pseudotime interactions (Fig. 7a). We identified 1932 genes (42%) affected by an interaction between the pseudotemporal trajectory and ER receptor status. The large percentage was expected given the heterogeneity of breast cancers and the strong stratification power of ER status in breast cancer subtyping^24^. Encouragingly (and to be expected), the Estrogen Receptor 1 (*ESR1*) was identified as one such gene. This positive control provided reassuring evidence that PhenoPath was discovering real interactions. Furthermore, the expression of fructose-1,6-biphosphatase (*FBP1*) and forkhead transcription factor C *FOXC1* also showed pseudotemporal dependence that was dependent on ER status (Figs. 6a and 7d). In the ER− regime, *FBP1* is upregulated along the trajectory while in the ER+ regime it is downregulated. Intriguingly, *FBP1* has been identified as a marker to distinguish ER+ from ER− subtypes and its expression has been shown to be negatively correlated with *SNAIL* as the Snail-G9a-Dnmt1 complex, is critical for E-cadherin promoter silencing, and required for the promoter methylation of FBP1 in basal-like breast cancer^37^ (Supplementary Fig. 18). Similarly, *FOXC1* which is known to be involved with ER*α* mediated action in breast cancer^38^ shows no regulation in the ER− regime yet is strongly upregulated in the ER+ case.

To complement this analysis, we performed a pathway enrichment analysis using Reactome^30^ to discover whether any of the top 20 interacting genes (by *β* value) converge on a cancer-related pathway. We found (at a FDR <5%) enrichment for Unfolded protein response and *ATF6α* activating chaperone genes. Previous studies have shown that knockouts of *ATF6α* blocked estrogen induction of the antiapoptotic chaperone BiP, which in turn inhibited ER-stimulated cell proliferation^39^. Therefore PhenoPath analysis suggests a relationship between the ER status of the tumour to the (vascular) growth via pathway-specific action mediated by *ATF6α*. The interaction gene set was further enriched for *TFAP2* family regulates transcription of growth factors and their receptors. *TFAP2* has previously been shown to directly interact with an estrogen receptor promoter^40^ and provides one of the key regulators of hormone responsiveness in breast cancers^41^. In particular, *TFAP2* has been shown experimentally to regulate some of the key genes we find as significant interactions, including *ESR1* and *FOXA1*^42^.

Many of these genes exhibit a convergence—they have markedly different expression at the beginning of the trajectory based on ER status yet converge towards the end. We derived a mathematical formula to infer such convergence points and calculated these for all genes showing significant interactions (see Supplementary Results for details). Remarkably, the vast majority converge towards the end of the trajectory (Fig. 7c), implying a common end-point in vascular development for both ER+ and ER− cancer subtypes. This effect can be seen in the trajectory plots in Fig. 6a, where the ER+ and ER− tumours converge at the end of their trajectories. This suggests that while there exist low levels of angiogenesis pathway activation, ER status dominates gene expression while as angiogenesis pathway activation increases it comes to dominate expression patterns over ER status. This finding might have implications for the application of angiogenesis inhibitors in breast cancer treatment.

## Discussion

PhenoPath provides a novel contribution to the existing arsenal of pseudotemporal analysis algorithms developed across a range of application areas including single cell ‘omics and cancer. Using a statistical model that allows for covariate-modulated pseudotemporal trajectories, PhenoPath generalises pseudotime analysis to a wider range of applications where genetic, phenotypic or environmental contexts may vary between samples and be influential in the trajectories. We have demonstrated its utility in an application to single-cell transcriptomics involving external stimuli and there is potential usage in high-throughput single-cell CRISPR experiments that are as yet unexplored^43,44^. We also demonstrated applications to The Cancer Genome Atlas using PhenoPath to model disease trajectories in colorectal and breast cancer. The trajectories identified were consistent with pre-existing knowledge concerning tumorigenesis in these diseases. Importantly, PhenoPath was able to identify covariate-pathway interactions that might be driving specific trajectory differences recovering known associations as well as novel genes. We showed that these behaviours cannot be readily determined with standard differential expression analyses without taking into account the latent disease progression. An assumption made by PhenoPath is that features evolve linearly with respect to pseudotime. We tested this assumption in a number of single cell data sets and found this approximation to be surprisingly accurate (Supplementary Results). However, it cannot be discounted that nonlinear effects may occur and checks should be conducted to verify that PhenoPath model fits are consistent with the data. Gaussian Processes offer a means of providing a more flexible nonlinear framework and further work in this area is anticipated. In summary, PhenoPath provides a powerful and scalable pseudotime analysis framework for modelling latent progression in a variety of experimental settings. Future work will expand the ability of PhenoPath to handle complex mixtures of continuous and discrete covariates in high-dimensional settings.

## Methods

We summarise the model specification and inference algorithms below. Further details are reported in Supplementary Methods.

### Statistical model

We begin with an *N* × *G* data matrix **Y** where *y*_*ng*_ denotes the *n*th entry in the *g*th column for *n* ∈ 1, …, *N* samples and *g* ∈ 1, …, *G* features. Such a matrix would correspond to the measurement of a dynamic molecular process that we might reasonably expect to show continuous evolution such as gene expression corresponding to a particular pathway. It is then trivial to learn a one-dimensional linear embedding that would be our “best guess” of such progression via a factor analysis model:

1 $$y_{ng} = \lambda _gz_n + \epsilon _{ng},\epsilon _{ng}\sim {\mathrm{N}}\left( {0,\tau _g^{ - 1}} \right),$$

where *z*_*n*_ is the latent measure of progression for sample *n* and *λ*_*g*_ is the factor loading for feature *g* which essentially describes the evolution of *g* along the trajectory.

However, it is conceivable that the evolution of feature *g* along the trajectory is not identical for all samples but is instead affected by a set of external covariates. Note that we expect such features to be “static” and should not correlate with the trajectory itself.

Introducing the *N* × *P* covariate matrix **X** with the entry in the *n*th row and *p*th column given by *x*_*np*_, we allow such measurements to perturb the factor loading matrix

2 $$\lambda _g \to \lambda _{ng} = \lambda _g + \mathop {\sum}\limits_{p = 1}^P {\kern 1pt} \beta _{pg}x_{np},$$

where *β*_*pg*_ quantifies the effect of covariate *p* on the evolution of feature *g*. Despite **Y** being column-centred we need to reintroduce gene and covariate-specific intercepts to satisfy the model assumptions, giving a generative model of the form

3 $$y_{ng} = \eta _g + \mathop {\sum}\limits_{p = 1}^P {\kern 1pt} \alpha _{pg}x_{np} + \left( {\lambda _g + \mathop {\sum}\limits_{p = 1}^P \beta _{pg}x_{np}} \right)z_n + \epsilon _{ng},\epsilon _{ng}\sim {\mathrm{N}}\left( {0,\tau _g^{ - 1}} \right).$$

Our goal is inference of *z*_*n*_ that encodes progression along with *β*_*pg*_ which is informative of novel interactions between continuous trajectories and external covariates. Consequently, we place a sparse Bayesian prior on *β*_*pg*_ of the form $\beta _{pg}\sim {\mathrm{N}}\left( {0,\chi _{pg}^{ - 1}} \right)$ where the posterior of *χ*_*pg*_ is informative of the model’s belief that *β*_*pg*_ is non-zero. The complete generative model is therefore given by

4 $$\begin{array}{l}\hskip -140pt\alpha _{pg}\sim {\mathrm{N}}\left( {0,\tau _\alpha ^{ - 1}} \right)\\ \hskip -135pt\lambda _g\sim {\mathrm{N}}\left( {0,\tau _\lambda ^{ - 1}} \right)\\ \hskip -128pt z_n\sim {\mathrm{N}}\left( {q_n,\tau _q^{ - 1}} \right)\\ \hskip -136pt\beta _{pg}\sim {\mathrm{N}}\left( {0,\chi _{pg}^{ - 1}} \right)\\ \hskip -115pt\chi _{pg}^{ - 1}\sim {\mathrm{Gamma}}({a_\beta ,b_\beta } )\\ \hskip -126pt\tau _g^{ - 1}\sim {\mathrm{Gamma}}(a,b)\\ \hskip -132pt\mu _g\sim {\mathrm{N}}\left( {0,\tau _\mu ^{ - 1}} \right)\\ \hskip -135pt\epsilon _{ng}\sim {\mathrm{N}}\left( {0,\tau _g^{ - 1}} \right)\\ y_{ng} = \mu _g + \mathop {\sum}\limits_p {\kern 1pt} \alpha _{pg}x_{np} + \left( {\lambda _g + \mathop {\sum}\limits_p {\kern 1pt} \beta _{pg}x_{np}} \right)z_n + \epsilon _{ng},\end{array}$$

where *τ*_*α*_, *τ*_*λ*_, *a*, *b*, *a*_*β*_, *b*_*β*_, *τ*_*q*_ are fixed hyperparameters and *q*_*n*_ encodes prior information about *z*_*n*_ if available but typically *q*_*n*_ = 0 ∀*i* in the uninformative case.

### Inference

We perform co-ordinate ascent mean field variational inference (see ref. ^45^) with an approximating distribution of the form

5 $$\begin{array}{l}\hskip -8ptq\left( {\left\{ {z_n} \right\}_{n = 1}^N,\{ {\mu _g} \}_{g = 1}^G,\{ {\tau _g} \}_{g = 1}^G,\{ {\lambda _g} \}_{g = 1}^G,} \right. \left. {\{ {\alpha _{pg}} \}_{g = 1,p = 1}^{G,P}\{ {\beta _{pg}} \}_{g = 1,p = 1}^{G,P}\{ {\chi _{pg}} \}_{g = 1,p = 1}^{G,P}} \right)\\ \hskip -8pt= \mathop {\prod}\limits_{n = 1}^N \underbrace {q_z\left( {z_n} \right)}_{{\mathrm{Normal}}}\mathop {\prod}\limits_{g = 1}^G \underbrace {q_\mu ( {\mu _g} )}_{{\mathrm{Normal}}}\underbrace {q_\tau ( {\tau _g} )}_{{\mathrm{Gamma}}}\underbrace {q_\lambda ( {\lambda _g} )}_{{\mathrm{Normal}}} \mathop {\prod}\limits_{p = 1}^P \underbrace {q_\alpha ( {\alpha _{pg}} )}_{{\mathrm{Normal}}}\underbrace {q_\beta ( {\beta _{pg}} )}_{{\mathrm{Normal}}}\underbrace {q_\chi ( {\chi _{pg}} )}_{{\mathrm{Gamma}}}\end{array}.$$

Due to the model’s conjugacy the optimal update for each parameter *θ*_*j*_ given all other parameters ***θ***_−*j*_ can easily be computed via

6 $$q_j^ \ast (\theta _j) \propto {\mathrm{exp}}\left\{ {{\bf{E}}_{ - j}[ {{\mathrm{log}}{\kern 1pt} p( {\theta _j|{\boldsymbol{\theta }}_{ - j},{\bf{X}},Y} )} ]} \right\},$$

where the expectation is taken with respect to the variational density over ***θ***_−*j*_. The precise form of the variational updates can be found in Supplementary Text.

### Ranking covariate-pathway interactions

For each gene *g* and covariate *p* we have *β*_*pg*_ that encodes the effect of *p* on the evolution of *g* along the trajectory **z**. We would like to identify interesting interactions for further analysis and follow-up. The variational approximation for *β*_*pg*_ is given by

7 $$q_{\beta _{pg}}\sim {\mathrm{N}}( {m_{\beta _{pg}},s_{\beta _{pg}}} ).$$

which after (approximately) maximising the ELBO will give estimates $\hat m_{\beta _{pg}}$ and $\hat s_{\beta _{pg}}$ for every gene and covariate. We classify or label an interaction as of interest if

8 $$\frac{{\left| {\hat m_{\beta _{pg}}} \right|}}{{\hat s_{\beta _{pg}}}} > \, k,$$

where *k* is a positive constant. In other words, the interaction is not of interest if *β*_*pg*_ = 0 falls within *k* posterior standard deviations of the posterior estimate of the mean of the interaction. This is equivalent to a decision theoretic loss criteria governing whether the true value for *β* lies in the tails of the posterior marginal or not.

### Data availability

We provide an R implementation of our method PhenoPath at https://bioconductor.org/packages/release/bioc/html/phenopath.html.

## Electronic supplementary material

Supplementary Information
