# Supplementary material for: Uncovering pseudotemporal trajectories with covariates from single cell and bulk expression data
Source: Nat Commun. 2018 Jun 22;9:2442. doi: 10.1038/s41467-018-04696-6 (PMC6015076; doi:10.1038/s41467-018-04696-6)
Supplement: Supplementary file 1 — Supplementary Information [file 41467_2018_4696_MOESM1_ESM.pdf]

Supplementary Information

Uncovering pseudotemporal trajectories with covariates from single cell and bulk expression data

Kieran R Campbell & Christopher Yau

May 10, 2018

Supplementary Methods

Data retrieval and preprocessing

Single cell analysis of primary mouse bone-marrow-derived dendritic cells

Single cell gene expression data for primary mouse bone-marrow-derived dendritic cells [1] were downloaded both as raw counts and TPM values from the *conquer* (consistent quantification of external rna-seq data) project [2] which uses *Salmon* [3]. A number of cells exhibited a low number of mapped reads and genes expressed (Supplementary Fig. 1) that were removed.

In the original publication [1], a number of cluster disrupted cells that exhibit high expression of *Serpinb6b* and low expression of *Lyz1* were identified. We therefore removed any cells whose $\log_2(\text{TPM}+1)$ expression of *Serpinb6b* exceeded 2.5 and in which *Lyz1* was not expressed (Supplementary Fig. 2).

Finally, there exhibited large variation in the number of genes expressed in each cell (Supplementary Fig. 3a) that correlated highly with the first principal component of the data (Supplementary Fig. 3B). Such an effect is known to be caused by underlying technical effects in single-cell RNA-seq data [4]. We therefore removed it using the `normaliseExprs` function in the `R` package `Scater` [5]. For the final analysis we retained 7,500 highly variable genes.

The Cancer Genome Atlas

For both COAD and BRCA studies, TPM matrices were retrieved from a recent transcript-level quantification of the entire TCGA study [6]. Clinical metadata, including the phenotypic covariates used in PhenoPath, were retrieved using the RTCGA `R` package [7]. Transcript level expression estimates were combined to gene level expression estimates using `Scater` [5].

A PCA visualisation of the COAD dataset (Supplementary Fig. 4a) showed two distinct clusters based on the plate of sequencing. Rather than try to correct such a large batch effect, we retained samples with a PC1 score of less than 0 and a PC3 score greater than -10, and removed any "normal" tumour types. For input to PhenoPath we used the 4,801 genes whose median absolute deviation in $\log(\text{TPM}+1)$ expression was greater than $\sqrt{\frac{1}{2}}$.

A PCA visualisation of the BRCA dataset (Supplementary Fig. 4b) showed a loosely dispersed outlier population that separated on the first and third principal components. We performed Gaussian mixture model clustering using the `R` package `mclust`[8], and removed samples designated as cluster 2 in Supplementary Fig. 4b, giving 1,135 samples for analysis. For input to PhenoPath we used the 4,579 genes whose variance in $\log(\text{TPM}+1)$ expression was greater than 1 and whose median absolute deviation was greater than 0.

Model Specification

The overall generative model for PhenoPath is given by the following Bayesian hierarchical construction,

$$
\begin{aligned}
\alpha_{pg} &\sim \mathcal{N}(0, \tau_\alpha^{-1}) \\
\lambda_g &\sim \mathcal{N}(0, \tau_\lambda^{-1}) \\
z_n &\sim \mathcal{N}(q_n, \tau_q^{-1}) \\
\beta_{pg} &\sim \mathcal{N}(0, \chi_{pg}^{-1}) \\
\chi_{pg}^{-1} &\sim \text{Gamma}(a_\beta, b_\beta) \\
\tau_g^{-1} &\sim \text{Gamma}(a, b) \\
\mu_g &\sim \mathcal{N}(0, \tau_\mu^{-1}) \\
\epsilon_{ng} &\sim \mathcal{N}(0, \tau_g^{-1}) \\
y_{ng} &= \mu_g + \sum_p \alpha_{pg} x_{np} + \left(\lambda_g + \sum_p \beta_{pg} x_{np} \right) z_n + \epsilon_{ng}
\end{aligned}
\tag{1}
$$

where the default values of the free hyperparameters are shown in Supplementary Table 2.

Co-ordinate ascent variational inference

We perform co-ordinate ascent mean field variational inference with an approximating distribution of the form

$$
\begin{aligned}
&q\left(\{z_n\}_{n=1}^N, \{\mu_g\}_{g=1}^G, \{\tau_g\}_{g=1}^G, \{\lambda_g\}_{g=1}^G, \{\alpha_{pg}\}_{g=1,p=1}^{G,P} \{\beta_{pg}\}_{g=1,p=1}^{G,P} \{\chi_{pg}\}_{g=1,p=1}^{G,P} \right) \\
&= \prod_{n=1}^N \underbrace{q_z(z_n)}_{\text{Normal}} \prod_{g=1}^G \underbrace{q_\mu(\mu_g)}_{\text{Normal}} \underbrace{q_\tau(\tau_g)}_{\text{Gamma}} \underbrace{q_\lambda(\lambda_g)}_{\text{Normal}} \prod_{p=1}^P \underbrace{q_\alpha(\alpha_{pg})}_{\text{Normal}} \underbrace{q_\beta(\beta_{pg})}_{\text{Normal}} \underbrace{q_\chi(\chi_{pg})}_{\text{Gamma}}
\end{aligned}
\tag{2}
$$

Due to the model's conjugacy the optimal update for each parameter θ_j given all other parameters $\boldsymbol{\theta}_{-j}$ can easily be computed via

$$
q_j^*(\theta_j) \propto \exp\left\{ \mathbf{E}_{-j} \left[\log p(\theta_j | \boldsymbol{\theta}_{-j}, \mathbf{X}, \mathbf{Y}) \right] \right\} \tag{3}
$$

where the expectation is taken with respect to the variational density over $\boldsymbol{\theta}_{-j}$. We must therefore calculate each conditional distribution followed by its expectation.

The conditional distributions are given below (where $\theta|\cdot$ can be interpreted as the conditional distribution of variable θ conditioned on *all* other variables and the data). For simplicity we assume the summation is obvious from the variable (i.e. $\sum_p \equiv \sum_{p=1}^P$, etc).

Conditional distribution of z

$$
z_n|\cdot \sim \mathcal{N}\left(\frac{\sum_g \tau_g k_{ng}(y_{ng} - \mu_g - \sum_p \alpha_{pg} x_{np}) + \tau_q q_n}{\sum_g \tau_g k_{ng}^2 + \tau_q}, [\sum_g \tau_g k_{ng}^2 + \tau_q]^{-1} \right) \tag{4}
$$

where $k_{ng} = \lambda_g + \sum_p \beta_{pg} x_{np}$.

Conditional distribution of α_{pg}

$$
\alpha_{pg}|\cdot \sim \mathcal{N}\left(\frac{\tau_g \sum_n (y_{ng} - \tilde{\mu}_{ng}^{\alpha_p}) x_{np}}{\tau_g \sum_n x_{np}^2 + \tau_\alpha}, [\tau_g \sum_n x_{np}^2 + \tau_\alpha]^{-1} \right) \tag{5}
$$

where

$$\tilde{\mu}_{ng}^{\alpha_p} = \mu_g + t_n(\lambda_g + \sum_{p'} \beta_{p'g} x_{np'}) + \sum_{p' \neq p} \alpha_{p'g} x_{np'} \tag{6}$$

in which $\sum_{p' \neq p}$ denotes the summation over 1 to P excluding p.

Conditional distribution of β_{pg}

$$\beta_{pg}|\cdot \sim \mathcal{N}\left(\frac{\tau_g \sum_n (y_{ng} - \tilde{\mu}_{ng}^{\beta_p}) x_{np} z_n}{\tau_g \sum_n z_n^2 x_{np}^2 + \chi_{pg}}, [\tau_g \sum_n z_n^2 x_{np}^2 + \chi_{pg}]^{-1}\right) \tag{7}$$

where

$$\tilde{\mu}_{ng}^{\beta_p} = \mu_g + z_n \lambda_g + \sum_{p'} \alpha_{p'g} x_{np'} + z_n \sum_{p' \neq p} \beta_{p'g} x_{np'} \tag{8}$$

Conditional distribution of τ_g

$$\tau_g|\cdot \sim \text{Gamma}\left(a + \frac{N}{2}, b + \sum_n \frac{(y_{ng} - \tilde{\mu}_{ng}^{\tau})^2}{2}\right) \tag{9}$$

where

$$\tilde{\mu}_{ng}^{\tau} = \mu_g + \sum_p \alpha_{pg} x_{np} + \left(\lambda_g + \sum_p \beta_{pg} x_{np}\right) \lambda_n. \tag{10}$$

Conditional distribution of χ_{pg}

$$\chi_{pg}|\cdot \sim \text{Gamma}\left(a_\beta + \frac{1}{2}, b_\beta + \frac{\beta_{pg}^2}{2}\right) \tag{11}$$

Conditional distribution of λ_g

$$\lambda_g|\cdot \sim \mathcal{N}\left(\frac{\tau_g \sum_n z_n (y_{ng} - \tilde{\mu}_{ng}^{\lambda})}{\tau_g \sum_n z_n^2 + \tau_\lambda}, [\tau_g \sum_n t_n^2 + \tau_\lambda]^{-1}\right) \tag{12}$$

where

$$\tilde{\mu}_{ng}^{\lambda} = \mu_g + \sum_p \alpha_{pg} x_{np} + \left(\sum_p \beta_{pg} x_{np}\right) z_n \tag{13}$$

Conditional distribution of μ_g

$$\mu_g|\cdot \sim \mathcal{N}\left(\frac{\tau_g \sum_n (y_{ng} - \nu_{ng})}{N\tau_g + \tau_\mu}, [N\tau_g + \tau_\mu]^{-1}\right) \tag{14}$$

where

$$\nu_{ng} = \sum_p \alpha_{pg} x_{np} + \left(\lambda_g + \sum_p \beta_{pg} x_{np}\right) z_n \tag{15}$$

Conditional expectation of z

$$
\begin{aligned}
\mathbf{E}_{-z_n}[\mu_{z_n}\tau_{z_n}] &= \sum_g \Big[\frac{a_{\tau_g}}{b_{\tau_g}}\Big(m_{\lambda_g} + \sum_p m_{\beta_{pg}} x_{np}\Big) \\
&\quad \times \Big(y_{ng} - m_{\mu_g} - \sum_p m_{\alpha_{pg}} x_{np}\Big)\Big] + \tau_q q_n \\
\mathbf{E}_{-z_n}[\tau_{z_n}] &= \sum_g \frac{a_{\tau_g}}{b_{\tau_g}}\Big(m^2_{\lambda_g} + s^2_{\lambda_g} + 2m_{\lambda_g}\sum_p m_{\beta_{pg}} x_{np} \\
&\quad + \sum_p (m^2_{\beta_{pg}} + s^2_{\beta_{pg}})x^2_{np} + \sum_{p,p':p\neq p'} m_{\beta_{pg}} m_{\beta_{p'g}} x_{np} x_{np'}\Big) + \tau_q
\end{aligned} \tag{16}
$$

where we have used the fact that

$$
\begin{aligned}
\mathbf{E}_{-z_n}[(\lambda_g + \sum_p \beta_{pg} x_{np})^2] &= \Big(m^2_{\lambda_g} + s^2_{\lambda_g} + 2m_{\lambda_g}\sum_p m_{\beta_{pg}} x_{np} \\
&\quad + \sum_p (m^2_{\beta_{pg}} + s^2_{\beta_{pg}})x_{np} + \sum_{p,p':p\neq p'} m_{\beta_{pg}} m_{\beta_{p'g}} x_{np} x_{np'}\Big)
\end{aligned} \tag{17}
$$

and for several variables that $\mathbf{E}_{-z_n}[\theta^2] = \text{Var}_{-z_n}[\theta] + \mathbf{E}_{-z_n}[\theta]^2$.

Conditional expectation of α_{pg}

$$
\begin{aligned}
\mathbf{E}_{-\alpha_{pg}}[\mu_{pg}\tau_{pg}] &= \frac{a_{\tau_g}}{b_{\tau_g}} \sum_n \Big(y_{ng} - m_{\mu_g} - m_{z_n}(m_{\lambda_g} + \sum_{p'} m_{\beta_{p'g}} x_{np}) \\
&\quad - \sum_{p'\neq p} m_{\alpha_{p'g}} x_{np'}\Big) x_{np} \\
\mathbf{E}_{-\alpha_{pg}}[\tau_{\alpha_{pg}}] &= \frac{a_{\tau_g}}{b_{\tau_g}} \sum_n x^2_{np} + \tau_\alpha
\end{aligned} \tag{18}
$$

Conditional expectation of β_{pg}

$$
\begin{aligned}
\mathbf{E}_{-\beta_{pg}}[\mu_{\beta_{pg}}\tau_{\beta_{pg}}] &= \frac{a_{\tau_g}}{b_{\tau_g}} \sum_n \Big[y_{ng} - m_{\mu_g} - \frac{m^2_{z_n} + s^2_{z_n}}{m_{z_n}} m_{\lambda_g} \\
&\quad - \sum_{p'} m_{\alpha_{p'g}} x_{np'} - \frac{m^2_{z_n} + s^2_{z_n}}{m_{z_n}} \sum_{p'\neq p} m_{\beta_{p'g}} x_{np}\Big] m_{z_n} x_{np} \\
\mathbf{E}_{-\beta_{pg}}[\tau_{\beta_{pg}}] &= \frac{a_{\tau_g}}{b_{\tau_g}} \sum_n (m^2_{z_n} + s^2_{z_n}) x^2_{np} + \frac{a_{\chi_{pg}}}{b_{\chi_{pg}}}
\end{aligned} \tag{19}
$$

where in both cases we have used the fact that $\mathbf{E}_{-\beta_{pg}}[z_n^2] = \text{Var}_{-\beta_{pg}}[z_n] + \mathbf{E}_{-\beta_{pg}}[z_n]^2$.

Conditional expectation of τ_g

$$
\begin{aligned}
\mathbf{E}_{-\tau_g}[a_{\tau_g}] &= a + \frac{N}{2} \\
\mathbf{E}_{-\tau_g}[b_{\tau_g}] &= b + \frac{1}{2}\sum_n f_{ng}
\end{aligned} \tag{20}
$$

where

$$
\begin{aligned}
f_{ng} &= \mathbf{E}_{-\tau_g}\left[\left(y_{ng} - \mu_g - \sum_p \alpha_{pg} x_{np} - \left(\lambda_g + \sum_p \beta_{pg} x_{np}\right) z_n\right)^2\right] \\
&= \mathbf{E}_{-\tau_g}\Bigg[\mu_g^2 + 2\mu_g \sum_p \alpha_{pg} x_{np} 2\mu_g z_n \lambda_g \\
&+ 2\mu_g z_n \sum_p \beta_{pg} x_{np} - 2y_{ng}\mu_g + +(\sum_p \alpha_{pg} x_{np})^2 \\
&2z_n \lambda_g \sum_p \alpha_{pg} x_{np} + 2z_n (\sum_p \alpha_{pg} x_{np})(\sum_p \beta_{pg} x_{np}) \\
&- 2y_{ng} \sum_p \alpha_{pg} x_{np} + z_n^2 \lambda_g^2 + 2\lambda_g z_n^2 \sum_p \beta_{pg} x_{np} \\
&- 2\lambda_g z_n y_{ng} + z_n^2 (\sum_p \beta_{pg} x_{np})^2 \\
&- 2y_{ng} z_n \sum_p \beta_{pg} x_{np} + y_{ng}^2\Bigg]
\end{aligned}
\tag{21}
$$

For this we require the identities

$$
\begin{aligned}
\mathbf{E}[\theta^2] &= \mathrm{Var}[\theta] + \mathbf{E}[\theta]^2 \\
\mathbf{E}[(\sum_p \gamma_{pg} x_{np})^2] &= \sum_p x_{np}^2 \mathbf{E}[\gamma_{pg}^2] + \sum_{p,p':p\neq p'} x_{np} x_{np'} \mathbf{E}[\gamma_{pg}\gamma_{p'g}]
\end{aligned}
\tag{22}
$$

This gives

$$
\begin{aligned}
f_{ng} = {} & m_{\mu_g}^2 + s_{\mu_g}^2 + \\
& + 2m_{\mu_g} \sum_p m_{\alpha_{pg}} x_{np} \\
& + 2m_{\mu_g} m_{z_n} m_{\lambda_g} \\
& + 2m_{\mu_g} m_{z_n} \sum_p m_{\beta_{pg}} x_{np} \\
& - 2y_{ng} m_{\mu_g} \\
& + \sum_p (m_{\alpha_{pg}}^2 + s_{\alpha_{pg}}^2) x_{np}^2 + \sum_{p,p':p \neq p'} m_{\alpha_{pg}} m_{\alpha_{p'g}} x_{np} x_{np'} \\
& + 2m_{z_n} m_{\lambda_g} \sum_p m_{\alpha_{pg}} x_{np} \\
& + 2m_{z_n} (\sum_p m_{\alpha_{pg}} x_{np})(\sum_p m_{\beta_{pg}} x_{np}) \\
& - 2y_{ng} \sum_p m_{\alpha_{pg}} x_{np} \\
& + (m_{z_n}^2 + s_{z_n}^2)(m_{\lambda_g}^2 + s_{\lambda_g}^2) \\
& + 2(m_{z_n}^2 + s_{z_n}^2) m_{\lambda_g} \sum_p m_{\beta_{pg}} x_{np} \\
& - 2m_{\lambda_g} m_{z_n} y_{ng} \\
& + (m_{z_n}^2 + s_{z_n}^2) \Big[\sum_p (m_{\beta_{pg}}^2 + s_{\beta_{pg}}^2) x_{np}^2 \\
& + \sum_{p,p':p \neq p'} m_{\beta_{pg}} m_{\beta_{p'g}} x_{np} x_{np'} \Big] \\
& - 2m_{z_n} y_{ng} \sum_p m_{\beta_{pg}} x_{np}) \\
& + y_{ng}^2
\end{aligned} \tag{23}
$$

Conditional expectation of χ_{pg}

$$
\begin{aligned}
\mathbf{E}_{-\chi_{pg}}[a_{\chi_{pg}}] &= a_\beta + \frac{1}{2} \\
\mathbf{E}_{-\chi_{pg}}[b_{\chi_{pg}}] &= b_\beta + \frac{1}{2} \left(m_{\beta_{pg}}^2 + s_{\beta_{pg}}^2 \right)
\end{aligned} \tag{24}
$$

where again we have used the fact that $\mathbf{E}_{-\chi_{pg}}[\beta_{pg}^2] = \text{Var}_{-\chi_{pg}}[\beta pg] + \mathbf{E}_{-\chi_{pg}}[\beta pg]^2$.

Conditional expectation of λ_g

$$
\begin{aligned}
\mathbf{E}_{-\lambda_g}[\mu_{\lambda_g} \tau_{\lambda_g}] &= \frac{a_{\tau_g}}{b_{\tau_g}} \sum_n m_{z_n} \Bigg(y_{ng} - m_{\mu_g} \\
& \quad - \sum_{p'} m_{\alpha_{p'g}} x_{np'} - \frac{m_{z_n}^2 + s_{z_n}^2}{m_{z_n}} (\sum_{p'} m_{\beta_{p'g}} x_{np'}) \Bigg) \\
\mathbf{E}_{-\lambda_g}[\tau_{\lambda_g}] &= \frac{a_{\tau_g}}{b_{\tau_g}} \sum_n (m_{z_n}^2 + s_{z_n}^2) + \tau_\lambda
\end{aligned} \tag{25}
$$

Conditional expectation of μ_g

$$\begin{aligned}\mathbf{E}_{-\mu_g}[\mu_{\mu_g}\tau_{\mu_g}] &= \frac{a_{\tau_g}}{b_{\tau_g}}\sum_n \Bigg(y_{ng} - \sum_{p'} m_{\alpha_{p'g}} x_{np'} \\ &\quad - m_{z_n}(m_{\lambda_g} + \sum_{p'} m_{\beta_{p'g}} x_{np'}) \Bigg) \\ \mathbf{E}_{-\mu_g}[\tau_{\mu_g}] &= \frac{a_{\tau_g}}{b_{\tau_g}} N + \tau_\mu\end{aligned} \tag{26}$$

To assess convergence of the CAVI algorithm we need to calculate the evidence lower bound (ELBO) at every iteration (or every i^{th} iteration). The ELBO is given by

$$\text{ELBO} = \mathbf{E}[\log p(\mathbf{Y}|\Theta)] + \mathbf{E}[\log p(\Theta)] - \mathbf{E}[\log q(\Theta)] \tag{27}$$

where all expectations are taken with respect to the approximating distribution $Q(\cdot)$ and Θ denotes the full parameter set. Note that we are implicitly conditioning on the data wherever appropriate, so $p(\mathbf{Y}|\Theta) \equiv p(\mathbf{Y}|\Theta, \mathbf{X})$. For this we require the result that if $\theta \sim \text{Gamma}(a, b)$ then $\mathbf{E}[\log \theta] = \phi(a) - \log b$ where ϕ is the digamma function $\phi(x) = \frac{\Gamma'(x)}{\Gamma(x)}$.

Derivation of $\mathbf{E}[\log p(\mathbf{Y}|\Theta)]$ We have

$$\log p(\mathbf{Y}|\Theta) = \sum_n \sum_g \log \mathcal{N}(y_{ng}|\mu_{ng}, \tau_g^{-1}) \tag{28}$$

where $\mu_{ng} = \mu_g + \sum_p \alpha_{pg} x_{np} + z_n \left(\lambda_g + \sum_p \beta_{pg} x_{np}\right)$. Then

$$\begin{aligned}\mathbf{E}[\log p(\mathbf{Y}|\Theta)] &\propto \sum_g \left[\frac{N}{2}\mathbf{E}[\log \tau_g] - \frac{\mathbf{E}[\tau_g]}{2} \sum_n \mathbf{E}[(y_{ng} - \mu_{ng})^2] \right] \\ &= \sum_g \left[\frac{N}{2}(\phi(a_{\tau_g}) - \log b_{\tau_g}) - \frac{a_{\tau_g}}{2 b_{\tau_g}} \sum_n f_{ng} \right]\end{aligned} \tag{29}$$

where f_{ng} is defined as above and we have dropped additive terms since we are only concerned by changes in the ELBO.

Derivation of $\mathbf{E}[\log p(\Theta)]$ We consider $\mathbf{E}[\log p(z_n)]$ which generalises to all parameters with Gaussian priors. We have

$$\begin{aligned}\mathbf{E}[\log p(z_n)] &= \mathbf{E}\left[\frac{1}{2}\log\frac{\tau_q}{2\pi} - \frac{\tau_q}{2}(z_n - q_n)^2\right] \\ &= \frac{1}{2}\log\frac{\tau_q}{2\pi} - \frac{\tau_q}{2}\mathbf{E}[z_n^2 - 2 z_n q_n + q_n^2] \\ &= \frac{1}{2}\log\frac{\tau_q}{2\pi} - \frac{\tau_q}{2}\left(m_{z_n}^2 + s_{z_n}^2 - 2 m_{z_n} q_n + q_n^2\right)\end{aligned} \tag{30}$$

Next consider $\mathbf{E}[\log p(\tau_g)]$ which generalises to all parameters with Gamma priors. We have

$$\begin{aligned}\mathbf{E}[\log p(\tau_g)] &\propto \mathbf{E}[\log(\tau_g^{a-1} e^{-\tau_g b})] \\ &= (a-1)\mathbf{E}[\log \tau_g] - b\mathbf{E}[\tau_g] \\ &= (a-1)(\phi(a_{\tau_g}) - \log b_{\tau_g}) - \frac{a_{\tau_g}}{b_{\tau_g}} b\end{aligned} \tag{31}$$

Thus the expression across all parameters up to a constant value is given by

$$
\begin{aligned}
\mathbf{E}[\log p(\Theta)] \propto & -\frac{\tau_q}{2}\sum_n (m_{z_n}^2 + s_{z_n}^2 - 2m_{z_n}q_n) \\
& -\frac{\tau_\mu}{2}\sum_g (m_{\mu_g}^2 + s_{\mu_g}^2) - \frac{\tau_\lambda}{2}\sum_g (m_{\lambda_g}^2 + s_{\lambda_g}^2) \\
& + \sum_g \left[(a-1)(\phi(a_{\tau_g}) - \log b_{\tau_g}) - \frac{a_{\tau_g}}{b_{\tau_g}} b \right] \\
& - \sum_p \sum_g \left[\frac{\tau_\alpha}{2}(m_{\alpha_{pg}}^2 + s_{\alpha_{pg}}^2) + \frac{a_{\chi_{pg}}}{2b_{\chi_{pg}}}(m_{\beta_{pg}}^2 + s_{\beta_{pg}}^2) \right. \\
& \left. - (a_\beta - 1)(\phi(a_{\chi_{pg}}) - \log b_{\chi_{pg}}) - \frac{a_{\chi_{pg}}}{b_{\chi_{pg}}} b_\beta \right]
\end{aligned}
\tag{32}
$$

Derivation of $\mathbf{E}[\log q(\Theta)]$ We consider $\mathbf{E}[\log q_z(z_n)]$ which naturally generalises to all parameters whose approximating distributions are Gaussian. We have

$$
\begin{aligned}
\mathbf{E}[\log q_z(z_n)] & \propto \mathbf{E}\left[-\frac{1}{2}\log s_{z_n}^2 - \frac{1}{2s_{z_n}^2}(z_n - m_{z_n})^2 \right] \\
& = -\frac{1}{2}\log s_{z_n}^2 - \frac{1}{2s_{z_n}^2}\mathbf{E}[z_n^2 - 2z_n m_{z_n} + m_{z_n}^2] \\
& = -\frac{1}{2}\log s_{z_n}^2 - \frac{1}{2s_{z_n}^2}\mathbf{E}[m_{z_n}^2 + s_{z_n}^2 - 2m_{z_n}^2 + m_{z_n}^2] \\
& \propto -\frac{1}{2}\log s_{z_n}^2
\end{aligned}
\tag{33}
$$

Similarly we consider $\mathbf{E}[\log q_\tau(\tau_g)]$ which generalises to all parameters whose approximating distribution is Gamma. We have

$$
\begin{aligned}
\mathbf{E}[\log q_\tau(\tau_g)] & = \mathbf{E}[a_{\tau_g}\log b_{\tau_g} + (a_{\tau_g} - 1)\log \tau_g - \tau_g b_{\tau_g} - \log\Gamma(a_{\tau_g})] \\
& = a_{\tau_g}\log b_{\tau_g} + (a_{\tau_g} - 1)(\phi(a_{\tau_g}) - \log b_{\tau_g}) - a_{\tau_g} - \log\Gamma(a_{\tau_g})
\end{aligned}
\tag{34}
$$

Summing this across all parameters gives

$$
\begin{aligned}
\mathbf{E}[\log q(\Theta)] = & -\frac{1}{2}\sum_n s_{z_n}^2 \\
& + \sum_g \Big(-\frac{1}{2}s_{\mu_g}^2 - \frac{1}{2}s_{\lambda_g}^2 + a_{\tau_g}\log b_{\tau_g} + (a_{\tau_g} - 1)(\phi(a_{\tau_g}) - \log b_{\tau_g}) \\
& - a_{\tau_g} - \log\Gamma(a_{\tau_g}) \Big) \\
& + \sum_g \sum_p \Big(-\frac{1}{2}s_{\alpha_{pg}}^2 - \frac{1}{2}s_{\beta_{pg}}^2 \\
& + a_{\chi_{pg}}\log b_{\chi_{pg}} + (a_{\chi_{pg}} - 1)(\phi(a_{\chi_{pg}}) - \log b_{\chi_{pg}}) - a_{\chi_{pg}} - \log\Gamma(a_{\chi_{pg}}) \Big)
\end{aligned}
\tag{35}
$$

Simulation Setup

We sought to quantify the extent to which having a joint model of pseudotimes and interactions aids identification of the interactions and the extent to which such interactions confound trajectory inference using traditional methods. Mathematically, PhenoPath infers the posterior distribution $p(\mathbf{z}, \boldsymbol{\beta}|\mathbf{Y})$ of the pseudotimes $\mathbf{z}$ and interaction parameters $\boldsymbol{\beta}$ given the data $\mathbf{Y}$. The two step procedure we compare against is analogous to first

inferring an estimate of the pseudotimes $\hat{\mathbf{z}}|\mathbf{Y}$ before inferring an estimate of the interaction parameters given the fixed pseudotimes $\hat{\boldsymbol{\beta}}|\hat{\mathbf{z}}, \mathbf{Y}$.

To do this we simulated RNA-seq data where a certain proportion of the genes exhibited different behaviour over pseudotime depending on the external covariate status. We then re-inferred the pseudotimes using a variety of algorithms (including PhenoPath), and performed post-hoc differential expression (DE) analysis testing for interaction effects between the trajectory and the covariate using common DE algorithms. In-depth details about the simulation, inference, and results are detailed below.

Mean function The first consideration is how we expect expression to change over pseudotime. We model this using non-linear sigmoid functions that have been successfully used to model single-cell RNA-seq evolution along trajectories previously (see e.g. [9]). Here, *mean* the expression of gene g in cell n at time t_n is modelled as $\mu(t_n, \mu_g^{(0)}, k_g, \delta_g) = 2\mu_g^{(0)}\sigma(k_g(t_n - \delta_g))$ where $\sigma(x) = (1 + \exp(-x))^{-1}$, and $\mu^{(0)}$, k, and δ are gene-specific parameters corresponding to the half-peak expression, rate of increase (or decrease) in expression over (pseudo)-time, and the point in pseudotime at which the rate of change is maximal respectively. Crucially, these parameters may be the same for all cells or dependent on the external covariate status of a cell, leading to interactions and ultimately different behaviour over pseudotime dependent on covariate status (Supplementary Fig. 5a). For each simulation, a certain proportion of genes were modelled as having different behaviour over pseudotime given the covariate status (the *interaction*). This mean function represents the behaviour of log-counts.

Noise model To simulate RNA-seq counts we used the negative binomial (NB) distribution that is the favoured noise model for RNA-seq counts (see e.g. [10, 11]). Here, the probability of observing y_{ng} counts in cell n and gene g is given by

$$p(y_{ng}|\mu_{ng}, \phi) = \frac{\Gamma(\phi + y_{ng})}{y_{ng}!\Gamma(\phi)} \left(\frac{\mu_{ng}}{\phi + \mu_{ng}}\right)^{y_{ng}} \left(\frac{\phi}{\phi + \mu_{ng}}\right)^{y_{ng}} \tag{36}$$

where ϕ is a size parameter that relates the variance to the mean via Var$[y_{ng}] = \mu_{ng} + \frac{\mu_{ng}}{\phi}$. For our simulations we created both a *low* noise models where $\phi = \mu_{ng}/3 + 1$ (corresponding to near-Poissonian noise, suggested by the authors of Polyester [12] for low-noise simulations) and a *high* noise model where $\phi = 1$ corresponding to overdispersed NB noise. The mean for each gene and cell used was $2^{\mu(t_n, \mu_g^{(0)}, k_g, \delta_g)} - 1$, where the function μ is defined above.

It is worth emphasising just how misspecified this model is with respect to the PhenoPath model:

1. The important gene specific parameters $(\boldsymbol{\lambda}, \boldsymbol{\alpha}, \boldsymbol{\beta})$ do not exist
2. The change in expression over time is modelled by non-linear sigmoid functions
3. The noise model is negative binomial, giving discrete, non-negative counts rather than the Gaussian noise model assumed by PhenoPath

Pseudotime inference Pseudotime inference was performed with PhenoPath, Monocle 2, DPT, and TSCAN. All algorithms were run with default parameter choices, with the exception of Monocle 2 and Wishbone. In Monocle 2 we set `norm_method = "none"` was used to ensure differing normalisation methods weren't responsible for different results. In the case of Wishbone, we found poor performance using the default parameters on our simulated data. After several attempts to get the best fit, we found the parameters that worked best were setting the number of nearest neighbours in the diffusion map calculation to 25% the total number of cells, the number of nearest neighbours in the trajectory analysis to 20% the number of cells, the number of way-points to 20% the number of cells, and using the first two diffusion components.

In all cases input data were the log-transformed counts normalised by total library size, with a pseudocount of 1 added to avoid divergence issues when $x \to 0$.

Differential expression Differential expression was performed with PhenoPath, Limma voom, ([13]) DESeq2, and MAST [14]. Raw counts were used for Limma voom and DESeq2 (as recommended), while log-normalised values were provided to MAST which is designed to work with $\log(\text{TPM} + 1)$. In all cases (with the exception of PhenoPath where differential expression testing is implicit in model inference), the models of `expression ~x:pseudotime` (interaction) was compared to the nested model `expression ~x + pseudotime` (no interaction). Default parameters were used for all methods.

AUC calculation Areas under the receiver-operator curves (AUCs) where calculated using the `AUC` `R` package. For differential expression analyses that report p-values, $1 - p-$value was used to rank genes for the likelihood of exhibiting a covariate interaction. For PhenoPath, we took the probability of observing 0 under the approximating distribution $q(m_{\beta_{pg}}, s_{\beta_{pg}})$ as inversely ranking genes for covariate interactions, and so $1-$ this value was used for input to the `AUC` function.

Simulation parameters The simulation parameters are described in Supplementary Table 3. In total, 6 different percentages of genes showing interactions were simulated across 2 noise regimes, 2 different cell numbers, with 40 replicates in each condition. Pseudotime inference using 4 different algorithms was then performed on this, with differential expression then performed using 4 different algorithms on the non PhenoPath pseudotimes. In total this gives

$$(6 \times 2 \times 2 \times 40) \times (1 + 3 \times 4) = 35,520$$

different differential expression workflows, representing a comprehensive benchmarking of PhenoPath. The overall simulation and inference workflow can be seen in Supplementary Fig. 5b.

Modelling zero-inflation In the simulations where we modelled zero inflation the overall strategy is to set a proportion $p \in \{0.05, 0.1, 0.2, 0.5, 0.8, 0.9\}$ of the non-zero counts to zero. In every dataset simulated (of raw counts), there is an existing proportion of genes b which are already zero due to being simulated using a negative-binomial likelihood. If there are NG counts in total (for N cells and G genes), the original proportion of zero counts was b, while the new number of zero counts is $bNG + (1 - b)pNG$. Therefore, we choose p of the $(1 - b)NG$ to be set to zero.

Zero inflation in scRNA-seq data is a non-random process that depends on the latent true expression. In particular, as the expression increases, the probability of observing a zero falls. This process can be well-modelled using logistic regression [15]. Using an unpublished scRNA-seq dataset of thymic epithelial cells, we computed the empirical dropout probability of each gene (proportion of zero reads) along with the sample mean, to which we fitted a logistic regression model in `R`, giving an intercept of 1.75 and expression coefficient of -0.04. Therefore, when choosing the $p(1 - b)NG$ counts to set to zero, the probability a particular count at level x is selected is given by $p_{\text{set to zero}} = \frac{1}{1+\exp(-(1.75-0.04x))}$.

Supplementary Results

Simulation study

We first compared accuracy of pseudotime inference across the range of conditions and pseudotime algorithms. The summarised results for 200 cells and the low noise case can be seen in Supplementary Fig. 5c. For a low fraction of genes exhibiting interactions between the covariate and latent space (5-10%) Monocle 2 appears to marginally outperform PhenoPath in the accuracy of pseudotime inference, possibly due to the non-linear nature of the simulated data and the linearity assumptions inherent to PhenoPath. We note however that PhenoPath does outperform two state-of-the-art nonlinear pseudotime inference methods within this range.

However, as the fraction of genes exhibiting interactions exceeds 10% the ability of PhenoPath to handle multiple covariates clearly becomes beneficial. The accuracy of PhenoPath's pseudotime inference is independent of the underlying fraction of genes exhibiting interactions between the covariate and latent space, while the performance of all other algorithms reduces drastically as this fraction increases. This pattern is evident across all benchmarking configurations studied (Supplementary Fig. 6). Since this fraction is unknown *a priori* we suggest it is necessary to use PhenoPath or a similar covariate-adjusted latent variable model to perform inference in such cases.

We then compared the ability of each workflow to detect covariate-trajectory interactions (i.e. does the status of the covariate affect how the gene changes over pseudotime?). For each dataset generated and each pseudotime fit for each dataset we performed differential expression analyses to detect covariate-trajectory interactions. For PhenoPath pseudotime we used PhenoPath's estimate of interaction significance as this is jointly estimated along with the pseudotimes. The accuracy of each workflow's ability to detect covariate-trajectory interactions was assessed using the area under the receiver-operator curve (AUC).

The results for 200 cells with the low noise regime and differential expression performed using Limma Voom can be seen in Supplementary Fig. 5d. On this simulated dataset (where the interactions have large effect sizes) PhenoPath and differential expression using Monocle 2 and TSCAN pseudotimes performs with near perfect AUCs (median AUC across 40 replicates = 1). However, as the fraction of genes exhibiting covariate-trajectory interactions increases, this performance reduces drastically for all workflows other than PhenoPath. Indeed, this pattern is replicated across all experiments and differential expression algorithms with both high noise (Supplementary Fig. 7) and low noise (Supplementary Fig. 8). As before, given the underlying fraction of genes that exhibits covariate-trajectory interactions is unknown *a priori* we suggest it is necessary to use PhenoPath for such analyses.

We were puzzled to discover some AUCs less than 0.5, apparently showing workflows performing *worse* than at random. We were initially worried that this was due to an error in implementation (though the fact that most workflows have AUCs near 1 when the pseudotimes are approximately correct acts as a positive control) so investigated one of the worst performing cases where pseudotime was inferred with Monocle 2 and differential expression performed with MAST.

In this dataset we examined one gene that had no interaction simulated but which the workflow identified has showing an interaction. The expression is shown against the true and inferred pseudotimes in Supplementary Fig. 9a&b respectively. It demonstrates that the inferred pseudotime in fact runs from samples with one covariate status into those of another. The fact that Monocle 2 attempts to find a trajectory with a degree of smoothness means these are joined at a "peak" in expression, resulting in the gradient of change over pseudotime of the gene for each covariate status being different and thus an interaction inferred. Essentially Monocle 2 has taken the pseudotime for one covariate status, flipped it, and rejoined the two separately where the expression is most similar.

We can then examine what happens to genes with covariate-dependent regulation along the trajectory, an example of which is shown in Supplementary Fig. 9c. The consequence of Monocle 2 reversing the pseudotimes for samples corresponding to one covariate status is that samples for both covariate statuses now have identical gradients over pseudotime, meaning no interaction is picked up using standard differential expression analyses. Therefore, we see how not only does performing separate pseudotime-DE analyses workflows for covariate-trajectory interactions reduce the power to detect interactions, it in fact means we can perform worse than by random guessing due to the characteristics of the trajectories inferred.

It is also worth emphasising the ease of which each analysis is performed. PhenoPath performs trajectory inference and detection of covariate-trajectory interactions with a call to a single R function. All other workflows we tested required the implementation of a bespoke bioinformatics pipeline.

Robustness to initialisation and hyperparameters

We sought to identify how robust PhenoPath is to the choice of hyperparameters and initialisation of the latent space z. An exhaustive analysis of all configurations is impractical, so we varied four different quantities and

compared the correlation in the inferred pseudotimes to those using the default PhenoPath values (section 1.2) for the mouse dendritic cell dataset [1]. Note that none of the values tested coincide the defaults. These four quantities were:

1. Percent change in the ELBO for optimisation to be considered converged, which we ranged across the values $10^{-3}, 10^{-4}, 10^{-6}$. Note that since PhenoPath implements co-ordinate ascent variational inference with exact updates for each parameter, this is the only free choice with respect to the variational inference procedure
2. The hyperparameter τ_α, with values $0.1, 2, 5$
3. The initialisation of z, which we took to be the capture times (scaled to have mean 0 standard deviation 1), initialisation from a different pseudotime algorithm (Monocle 2), and the first principal component of the data corrupted by additive $\mathcal{N}(0, 1)$ noise
4. The quantity $\frac{a_\beta}{b_\beta}$, which controls the mean of the Gamma prior on χ, which in turn controls the shrinkage on the interaction coefficients β. The variance of this distribution was fixed to 10.

In total this gives $3^4 = 81$ hyperparameter/initialisation conditions that were used to fit the mouse dendritic cells dataset [1]. The pseudotimes reported by these fits were correlated with the pseudotimes using the default PhenoPath values, the results of which can be seen in Supplementary Fig. 10. Across all parameter combinations, the *minimum* correlation to the default values is 0.9993, suggesting PhenoPath is highly robust to the initialisation of the latent space, hyperparameters, and variational inference algorithm.

Single-cell pseudotime is approximately linear

One common theme in many pseudotime inference algorithms is the emphasis on learning a *non*-linear manifold embedded in the high dimensional space (see e.g. [16, 17]) which could in theory decrease the accuracy of trajectories inferred with PhenoPath that assumes linear changes in expression over (pseudo-)time. Since the pseudotimes are always unobserved it is impossible to precisely quantify the true non-linearity of the "pseudotemporal manifold". However, we can fit trajectories and compare the results with those inferred using the "nonlinear" algorithms.

We assembled four single-cell RNA-seq datasets previously used in pseudotime analyses ([18, 19, 20, 21]) and for each fitted pseudotimes using Monocle 2 ([16]), Diffusion Pseudotime (DPT, [17]), and TSCAN ([22]) and examined the correlation to the first principal component of the data (computed using `prcomp` in `R`), the results of which can be seen in Supplementary Fig. 11a.

In the majority of cases the correlation of the pseudotime inferred to the first principal component of the dataset exceeds 0.8, with the minimum value still greater than 0.5. We further fitted a linear model (using `lm` in `R`) for each gene as a function of the pseudotime derived for each algorithm in each dataset and examined the proportion of the transcriptome that has a significant linear trend (where *significant* is defined as the Benjamini-Hochberg corrected p-value for the linear coefficient being less than 0.05). Supplementary Fig. 11b shows that for all datasets and algorithms at least half the transcriptome exhibits an approximately linear trend.

PhenoPath is robust to highly variable gene selection

The trajectory inferred by pseudotime algorithms is obviously dependent on the genes used. A common approach is to select a subset of highly variable genes (HVGs) ([23]), though the precise number of HVGs to use is of course subjective and will affect the pseudotime inferred.

We tested the extent to which PhenoPath is robust to the number of HVGs used as input to the algorithm. For the three datasets we studied we varied the number of HVGs used and examined the correlation to the latent space derived using the 1000 HVGs, the results of which can be seen in Supplementary Fig. 12.

Across each dataset we PhenoPath was generally robust to the number of HVGs selected, with the lowest reported correlation of 0.45. We repeated this exercise using one of the leading pseudotime methods (Monocle 2, [16]) to understand the extent to which such variability is inherent to all pseudotime algorithms (note that the correlation reported in this case is to the Monocle 2 pseudotime on 1000 HVGs). We found that Monocle 2 was also sensitive to the set of HVGs included in the analysis.

Demonstration of PhenoPath with categorical variables

One of PhenoPath's strengths is its ability to work with arbitrary design matrices incorporating binary, categorical, or continuous covariates. For this we simply construct a design matrix using the `model.matrix` command in `R` (with *no* intercept as this is handled by the λ parameter) and pass this in as x to the model, e.g. for three categories we would make the design matrix with

```
x <- factor(sample(1:3, N, replace = TRUE))
x_mat <- model.matrix(~ 0 + x)
```

then call PhenoPath with

```
fit <- phenopath(Y, x_mat)
```

for some expression matrix `Y`. We performed a small simulation study on toy data to demonstrate PhenoPath's ability to handle such input matrices. We simulated data for 80 genes and 100 cells from the PhenoPath mean function, each of which belonged to one of three different "types", and added $\mathcal{N}(0, 1)$ noise. PCA plots of the expression data coloured by pseudotime and covariate status may be seen in Supplementary Fig. 13. We subsequently re-inferred using the `phenopath()` function in `R` passing in a design matrix as described above with all other parameters left as default.

The results can be seen in Supplementary Fig. 14. Supplementary Fig. 14a shows that inference of the latent z values is highly accurate when compared to the true z values. We further examined the estimated β values compared to the true simulated values, which exhibits high correlation with slight shrinkage due to the automatic relevance determination (ARD) prior. However, the significance test for this is well calibrated, exhibiting high sensitivity and specificity.

Note that categorical covariates must be converted to a one-hot encoding matrix that can substantially increase the number of parameters to be inferred. If a categorical variable has M levels then $M \times G$ additional variables are introduced for G genes.

Comparison to fitting separate pseudotimes for each covariate status

An alternative approach to that of the PhenoPath model is to split the samples based on covariate-status and perform pseudotime inference separately on each before combining the results post-hoc. There are several downsides to such an approach. Examining each set of cells separately leads to smaller numbers of samples per test and therefore a reduction in power to detect interactions. Furthermore, if the covariate is continuous one would have to resort to arbitrary binning of samples to perform such an analyses. This becomes even more burdensome if multiple covariates are present. For example, if x_1 has levels A and B and x_2 has levels C and D we would have to consider four different groups of samples (AC, AD, BC, BD) - and fit the pseudotimes separately for each. The number of groups grows exponentially in the number of factors, meaning many groups will actually have few or no samples in it. PhenoPath circumvents all of these issues by default as part of its integrated model.

Further subtle issues arise with respect to biological interpretation. Upon splitting the samples, how do you know the inferred pseudotimes correspond to the same biological process? A recent study has looked into this (preprinted after our original submission, [24]) that uses dynamic time warping to "align" multiple trajectories. This approach will not work if the covariate is continuous, becomes combinatorially hard with the number of levels if x is categorical, and is taken care of by default in the PhenoPath model by modelling a common process z for all samples.

To demonstrate some of the downsides to such a split-analysis approach we took the mouse dendritic cells dataset [1] and performed pseudotime inference separately for each stimulant status (LPS and PAM) using the 5000 HVG. Our first observation was that the R^2 to capture time using Monocle was 0.64 and 0.55 for LPS and PAM respectively, compared to 0.70 for PhenoPath.

It is worth considering the difficulties in interpretation of interactions when departing from linear models. Consider the example of the gene *Tnf* from the mouse dendritic cells dataset [1], that is downregulated under LPS stimulation but upregulated under PAM. If we split the dataset and perform DE analysis, *Tnf* will be significant under both, from which we could naively conclude there is no difference in regulation between LPS and PAM. If using the default B-spline basis for Monocle 2 we could then turn to the coefficients to see if there is any difference between them. However, the B-spline basis is nonparametric in nature making coefficients hard to compare between two different datasets in an intuitive manner. We therefore restrict this analysis to linear changes in expression over pseudotime, fitting models of the form $y = \beta_{\text{Stimulant}} z$ and comparing $\hat{\beta}_{\text{LPS}} - \hat{\beta}_{\text{PAM}}$ to test for interactions.

We performed this analysis on the mouse dendritic cells dataset [1] as described above and compared the inferred $\hat{\beta}_{\text{LPS}} - \hat{\beta}_{\text{PAM}}$ to the PhenoPath β interaction parameters (Supplementary Fig. 15a), finding virtually no correlation. After further analyses we discovered that the pseudotime for LPS cells was orientated "backwards" with respect to true time (which is fine since all pseudotimes are equivalent up to a parity transformation), and upon "re-orientating" it and performing the analysis again we found the estimates in good agreement (Supplementary Fig. 15a).

While correcting the initially wrong orientation was easy given we had ground-truth capture times, such a step is almost impossible if we do not. Given the orientation is essentially random, there is a 50% chance that one trajectory will be orientated the wrong way compared to another, which obviously increases as the number of trajectories we are trying to integrate does too. Furthermore we cannot appeal to steps such as identifying which orientation gives the most coefficients in common, as it is precisely differences here we are searching for in the first place. Finally, we note that while we can examine $\hat{\beta}_{\text{LPS}} - \hat{\beta}_{\text{PAM}}$ to rank interactions this does not provide a means of testing for *significant* interactions without complex mathematics to transform the confidence intervals. PhenoPath circumvents all these issues by using an integrated model.

Effect of zero-inflation (dropout) on PhenoPath

We wished to quantify the effect of single-cell dropout on PhenoPath when PhenoPath was used with single-cell data. Using the same simulation scheme as before, we simulated (with the "high" noise regime) datasets where a fraction $p \in \{0.05, 0.1, 0.2, 0.5, 0.8, 0.9\}$ of the non-zero counts are set to zero, with 40 replicates in each condition (see section 1.4). We then re-inferred the pseudotimes using PhenoPath.

The results can be seen in Supplementary Fig. 16. The ability of PhenoPath to infer pseudotime is largely unaffected by single-cell dropout until $> 90\%$ of non-zero counts are set to zero. We believe this is due to the non-random nature of single-cell dropout - lowly expressed genes are more likely to be zeroed than highly expressed genes, so in general inference will be robust to say a count of $\log(\text{TPM} + 1) = 1$ being set to zero than a count of $\log(\text{TPM} + 1) = 10$ being set to zero.

BRCA Survival Analysis

We fitted a stratified (ER status) Cox proportional hazards model to the overall survival data for 720 TCGA BRCA patients with survival and expression data using patient age at onset and PhenoPath pseudotime as covariates. This survival analysis indicated that the model coefficient associated with the pseudotime contribution was significant ($p = 0.0032$). Analysis of deviance between nested models, with and without pseudotime as a covariate, indicated that the performance of the more complex model that includes pseudotime produces a better fit to the survival data $p = 0.004124$. Proportional hazards tests and diagnostics based on weighted residuals was performed to confirm that the proportion hazards assumption was not violated. Supplementary

Fig. 17 suggests that under both ER+ and ER-, increasing pseudotime progression leads to reduced overall survival.

Identifying crossover points in BRCA

In PhenoPath we model gene expression evolving along the trajectories separately for each phenotype (or covariate) considered. Unless the gradient of change along the trajectory is exactly equal for both phenotypes (i.e. $\beta = 0$ exactly), the gene expression will cross at a given point in the trajectory.

Inference of this point would allow us to identify sections of the trajectory not affected by the covariate and consequently sections of the trajectory that are. This is important as if the crossover point occurs towards the beginning of the trajectory, it would mean gene expression is similar at the beginning but diverges as we move along the trajectory. Similarly, if the crossover points occur towards the end of the trajectory, it would imply the expression profiles for the two phenotypes are different at the beginning of the trajectory, but converge as the trajectory progresses. An interpretation of this would be that the effect on expression from the trajectory slowly dominates over the effect of phenotypes on the trajectory.

It is important to note that the latent trajectory values loosely follow a $\mathrm{N}(0, 1)$ distribution. This means the 'middle' of the trajectory is any value around zero, values of -1 or less could be thought of as the 'beginning' while values greater than 1 may be thought of as the 'end'. Crucially, we can derive an analytical expression from the PhenoPath parameters for the crossover point z^*.

The condition for the crossover point is that the predicted expression for each phenotype is identical. Therefore (in the context of BRCA cancer)

$$y_g^{\text{ER+}}(z_g^*) = y_g^{\text{ER-}}(z_g^*) \tag{37}$$

which leads to the condition

$$\alpha_g x_{\text{ER+}} + (c_g + \beta_g x_{\text{ER+}})\, z_g^* = \alpha_g x_{\text{ER-}} + (c_g + \beta_g x_{\text{ER-}})\, z_g^* \tag{38}$$

which is in turn solved by

$$z_g^* = -\frac{\alpha_g}{\beta_g}. \tag{39}$$

We fitted the crossover points z^* for all *significant* genes in the BRCA dataset. We find that the vast majority of the crossover times z^* occur towards the end of the trajectory, with a median value of around 0.4. In other words, at the beginning of the trajectory most genes are differentially expressed based on ER status, while as the trajectory progresses it comes to dominate at the gene expression converges.

References

[1] Alex K Shalek, Rahul Satija, Joe Shuga, John J Trombetta, Dave Gennert, Diana Lu, Peilin Chen, Rona S Gertner, Jellert T Gaublomme, Nir Yosef, Schraga Schwartz, Brian Fowler, Suzanne Weaver, Jing Wang, Xiaohui Wang, Ruihua Ding, Raktima Raychowdhury, Nir Friedman, Nir Hacohen, Hongkun Park, Andrew P May, and Aviv Regev. Single-cell RNA-seq reveals dynamic paracrine control of cellular variation. *Nature*, 510(7505):363–369, 19 June 2014.

[2] Charlotte Soneson and Mark D Robinson. Bias, robustness and scalability in differential expression analysis of single-cell rna-seq data. *bioRxiv*, page 143289, 2017.

[3] Rob Patro, Geet Duggal, Michael I Love, Rafael A Irizarry, and Carl Kingsford. Salmon provides fast and bias-aware quantification of transcript expression. *Nature Methods*, 14(4):417–419, 2017.

[4] Stephanie C Hicks, F William Townes, Mingxiang Teng, and Rafael A Irizarry. Missing data and technical variability in single-cell rna-sequencing experiments. *bioRxiv*, page 025528, 2017.

[5] Davis J McCarthy, Kieran R Campbell, Aaron TL Lun, and Quin F Wills. Scater: pre-processing, quality control, normalization and visualization of single-cell rna-seq data in r. *Bioinformatics*, 33(8):1179–1186, 2017.

[6] P J Tatlow and Stephen R Piccolo. A cloud-based workflow to quantify transcript-expression levels in public cancer compendia. *Sci. Rep.*, 6:39259, 16 December 2016.

[7] Marcin Kosinski and Przemyslaw Biecek. *RTCGA: The Cancer Genome Atlas Data Integration*, 2016. R package version 1.4.0.

[8] C Fraley, A E Raftery, T B Murphy, and L Scrucca. mclust version 4 for r: Normal mixture modeling for Model-Based clustering, classification, and density estimation. 2012. *University of Washington: Seattle*, 2012.

[9] Kieran R Campbell and Christopher Yau. switchde: inference of switch-like differential expression along single-cell trajectories. *Bioinformatics*, 23 December 2016.

[10] Michael I Love, Wolfgang Huber, and Simon Anders. Moderated estimation of fold change and dispersion for RNA-seq data with DESeq2. *Genome Biol.*, 15(12):550, 2014.

[11] Mark D Robinson, Davis J McCarthy, and Gordon K Smyth. edger: a bioconductor package for differential expression analysis of digital gene expression data. *Bioinformatics*, 26(1):139–140, 1 January 2010.

[12] Alyssa C Frazee, Andrew E Jaffe, Ben Langmead, and Jeffrey T Leek. Polyester: simulating RNA-seq datasets with differential transcript expression. *Bioinformatics*, 31(17):2778–2784, 1 September 2015.

[13] Charity W Law, Yunshun Chen, Wei Shi, and Gordon K Smyth. voom: Precision weights unlock linear model analysis tools for RNA-seq read counts. *Genome Biol.*, 15(2):R29, 3 February 2014.

[14] Greg Finak, Andrew McDavid, Masanao Yajima, Jingyuan Deng, Vivian Gersuk, Alex K Shalek, Chloe K Slichter, Hannah W Miller, M Juliana McElrath, Martin Prlic, Peter S Linsley, and Raphael Gottardo. MAST: a flexible statistical framework for assessing transcriptional changes and characterizing heterogeneity in single-cell RNA sequencing data. *Genome Biol.*, 16:278, 10 December 2015.

[15] Peter V Kharchenko, Lev Silberstein, and David T Scadden. Bayesian approach to single-cell differential expression analysis. *Nature methods*, 11(7):740, 2014.

[16] Xiaojie Qiu, Qi Mao, Ying Tang, Li Wang, Raghav Chawla, Hannah A Pliner, and Cole Trapnell. Reversed graph embedding resolves complex single-cell trajectories. *Nat. Methods*, 21 August 2017.

[17] Laleh Haghverdi, Maren Büttner, F Alexander Wolf, Florian Buettner, and Fabian J Theis. Diffusion pseudotime robustly reconstructs lineage branching. *Nat. Methods*, 13(10):845–848, October 2016.

[18] Fan Zhou, Xianlong Li, Weili Wang, Ping Zhu, Jie Zhou, Wenyan He, Meng Ding, Fuyin Xiong, Xiaona Zheng, Zhuan Li, Yanli Ni, Xiaohuan Mu, Lu Wen, Tao Cheng, Yu Lan, Weiping Yuan, Fuchou

Tang, and Bing Liu. Tracing haematopoietic stem cell formation at single-cell resolution. *Nature*, 533(7604):487–492, 26 May 2016.

[19] Ben W Dulken, Dena S Leeman, Stéphane C Boutet, Katja Hebestreit, and Anne Brunet. Single-Cell transcriptomic analysis defines heterogeneity and transcriptional dynamics in the adult neural stem cell lineage. *Cell Rep.*, 18(3):777–790, 17 January 2017.

[20] Li-Fang Chu, Ning Leng, Jue Zhang, Zhonggang Hou, Daniel Mamott, David T Vereide, Jeea Choi, Christina Kendziorski, Ron Stewart, and James A Thomson. Single-cell RNA-seq reveals novel regulators of human embryonic stem cell differentiation to definitive endoderm. *Genome Biol.*, 17(1):173, 17 August 2016.

[21] Cole Trapnell, Davide Cacchiarelli, Jonna Grimsby, Prapti Pokharel, Shuqiang Li, Michael Morse, Niall J Lennon, Kenneth J Livak, Tarjei S Mikkelsen, and John L Rinn. The dynamics and regulators of cell fate decisions are revealed by pseudotemporal ordering of single cells. *Nat. Biotechnol.*, 32(4):381–386, April 2014.

[22] Zhicheng Ji and Hongkai Ji. TSCAN: Pseudo-time reconstruction and evaluation in single-cell RNA-seq analysis. *Nucleic Acids Res.*, 44(13):e117, 27 July 2016.

[23] Rhonda Bacher and Christina Kendziorski. Design and computational analysis of single-cell RNA-sequencing experiments. *Genome Biol.*, 17:63, 7 April 2016.

[24] Andrew Butler and Rahul Satija. Integrated analysis of single cell transcriptomic data across conditions, technologies, and species. *bioRxiv*, page 164889, 2017.

Supplementary Figures

List of Figures

1 **Quality control for single cell mouse dendritic cell data.** Total number of genes expressed (`total_features`) vs total number of reads mapping (`total_counts`) for mouse dendritic cells [1]. 22

2 **Data filtering for disrupted mouse dendritic cells.** Expression of *Serpbinb6b* and *Lyz1* across mouse dendritic cells [1] 23

3 **Technical effects in single cell mouse dendritic cell data. A** Histogram of number of genes expressed across all mouse dendritic cells. **B** The correlation of the total number of genes expressed to principal components. 24

4 **Principal Components Analysis of batch effects in TCGA COAD and BRCA data.** PCA representations of the COAD (**a**) and BRCA (**b**) datasets, coloured by sequenced plate and GMM cluster assignment respectively. 25

5 **Benchmarking PhenoPath performance on simulated data sets.** Comprehensive benchmarking of PhenoPath demonstrates the necessity of a joint model of covariates and latent progression. **a** Genes were either simulated as evolving identically along pseudotime regardless of covariate status (*no interaction*) or coming from one of four interacting types. **b** Multiple datasets with differing transcriptional noise were quantified by different pseudotime algorithms and differential expression models leading to 35,520 different workflows. **c** Median Kendall's-τ to true pseudotime for 200 cells in the low noise case. For the smallest fraction of genes exhibiting interactions Monocle 2 performs best, though PhenoPath maintains the best overall performance as the fraction of interactions increases. Error bars show the 95% interval over 40 replications. **d** Area under the curve for detecting interactions shows good performance for Monocle 2, PhenoPath and TSCAN with a low fraction of genes interacting for 200 cells in the low noise case with Limma Voom used for DE. However, as the fraction of interactions increases the performance of the other algorithms quickly decreases, with some performing worse than would be expected at random. 26

6 **Comparison of pseudotime algorithm performance in the presence of interactions.** Performance of various pseudotime algorithms as a function of the fraction of genes exhibiting interactions, for both the low and high noise simulation cases and with 200 and 500 cells. . . 27

7 **Comparison of joint pseudotime and differential expression pipeline performance for detecting the presence of interactions.** AUCs for detecting covariate-trajectory interactions for all simulated datasets and pseudotime algorithms with differential expression performed using Limma Voom, DESeq2, MAST, and Monocle 2 for the *high* noise regime. DESeq2 returned `NA` p-values for many fits due to high magnitude outliers driven by the large overdispersion present (TSCAN only returns pseudotimes for a subset of samples which we assume resulted in exclusion of the "unusual" samples). 28

8 **Comparison of differential expression analysis algorithm performance for detecting the presence of interactions.** AUCs for detecting covariate-trajectory interactions for all simulated datasets and pseudotime algorithms with differential expression performed using Limma Voom, DESeq2, MAST, and Monocle 2 for the *low* noise regime. 29

9 **Comparison of standard workflow performances in the presence of interactions.** A standard pseudotime-DE workflow can lead to severe mis-classification of covariate-trajectory interactions. **a** Simulated expression of a gene that exhibits covariate-independent regulation over pseudotime, as a function of simulated pseudotime. **b** The same gene's expression as a function of the Monocle-inferred pseudotime. **c** Simulated expression of a gene that exhibits covariate-dependent regulation over pseudotime, as a function of simulated pseudotime. **d** The same gene's expression as a function of the Monocle-inferred pseudotime. 30

10 **Analysis of PhenoPath performance using different initialisations.** A total of $3^4 = 81$ different initialisations were used for PhenoPath fitted to the mouse dendritic cells dataset [1], across a range of parameter values for the threshold change in the ELBO to decide convergence (**a**), the hyperparameter τ_α (**b**), the initialisation of the latent space (**c**), and the mean of the Gamma prior distribution on the ARD precisions on β (**d**). Boxplot lower and upper hinges correspond to the first and third quantile. Upper and lower whiskers correspond to 1.5 times the inter-quartile range from the hinge. Data outside this range is considered an outlier. . . . 31

11 **Comparison of non-linear pseudotime algorithms with principal components analysis.** Single-cell pseudotime is approximately linear. **a** The correlation to the first principal component of the data across the algorithms Monocle 2, DPT, and TSCAN for four single-cell datasets. **b** The proportion of the transcriptome that exhibits a significant linear trend for each pseudotime algorithm and dataset (FDR = 5%). 32

12 **Comparison of pseudotime estimation as a function of varying data input.** Correlation of the inferred latent space (z) for PhenoPath using a given number of highly variable genes across the three datasets studied to the result using 1000 highly variable genes (blue). In general this is more robust than the same analysis using Monocle 2 (red). 33

13 **Simulated data illustration.** PCA plot of the simulated toy dataset, coloured by pseudotime (**a**) and covariate status (**b**). 34

14 **PhenoPath analysis of categorical covariate data.** **a** The inferred pseudotime against the simulated pseudotime. **b** The inferred β interaction parameters compared to the true values. 34

15 **Comparison of split-data analysis with PhenoPath integration.** **A** Comparison of $\hat{\beta}_{\text{LPS}} - \hat{\beta}_{\text{PAM}}$ values to PhenoPath β interaction coefficients in the intial analysis. **B** The same comparison after "re-orientating" the LPS trajectory. 35

16 **Effect of zero-inflation (dropout) on PhenoPath**. Boxplots showing correlation to true pseudotime across simulations with different percentage of zero counts. Lower and upper hinges correspond to the first and third quantile. Upper and lower whiskers correspond to 1.5 times the inter-quartile range from the hinge. Data outside this range is considered an outlier. Setting non-zero measurements to zero in simulations does not significantly affect the accuracy of PhenoPath. 36

17 **TCGA BRCA Survival Analysis.** A stratified (by ER status) Cox Proportional Hazards models was fit using PhenoPath derived pseudotimes as a covariate. 37

18 **ER status marker expression.** FBP1 expression is inversely correlated with Snail in ER- breast cancers but shows no dependence in ER+ breast cancers. 38

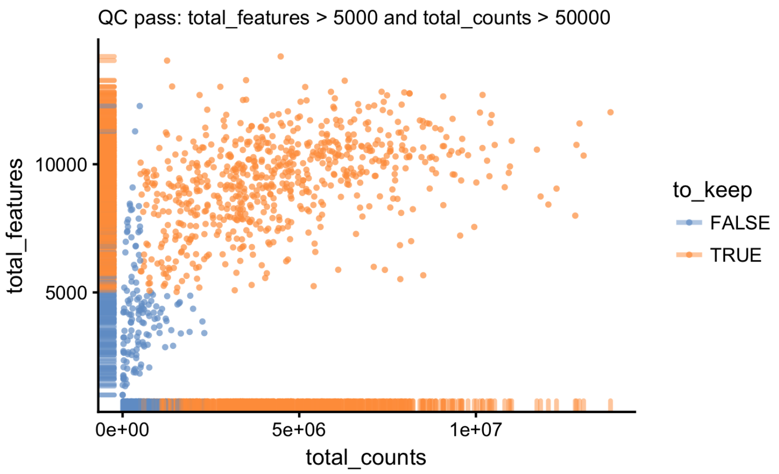

Supplementary Figure 1: Quality control for single cell mouse dendritic cell data. Total number of genes expressed (`total_features`) vs total number of reads mapping (`total_counts`) for mouse dendritic cells [1].

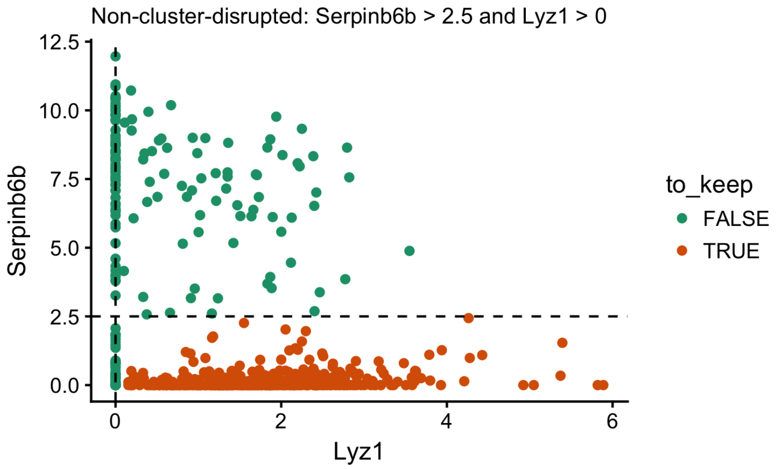

Supplementary Figure 2: Data filtering for disrupted mouse dendritic cells. Expression of *Serpbinb6b* and *Lyz1* across mouse dendritic cells [1]

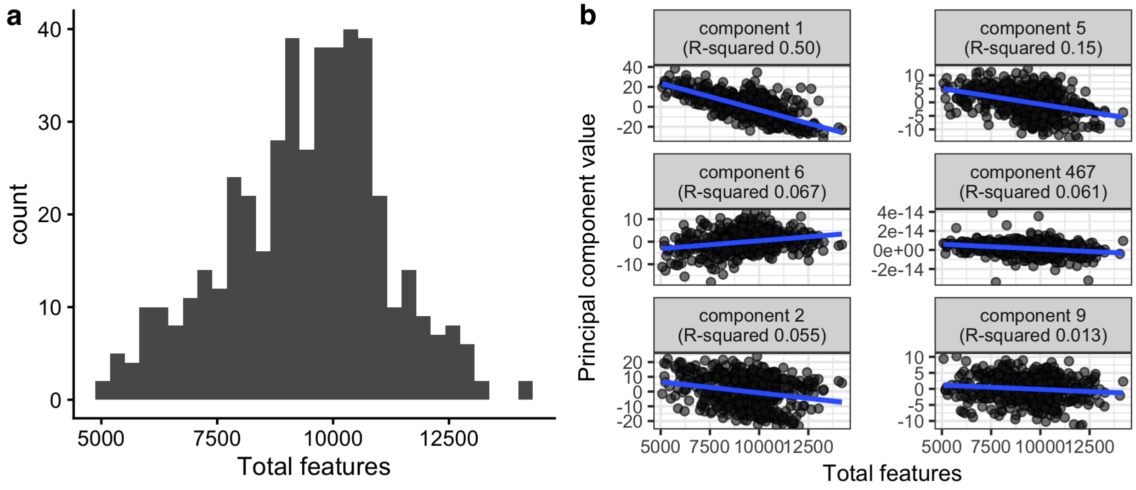

Supplementary Figure 3: Technical effects in single cell mouse dendritic cell data. **A** Histogram of number of genes expressed across all mouse dendritic cells. **B** The correlation of the total number of genes expressed to principal components.

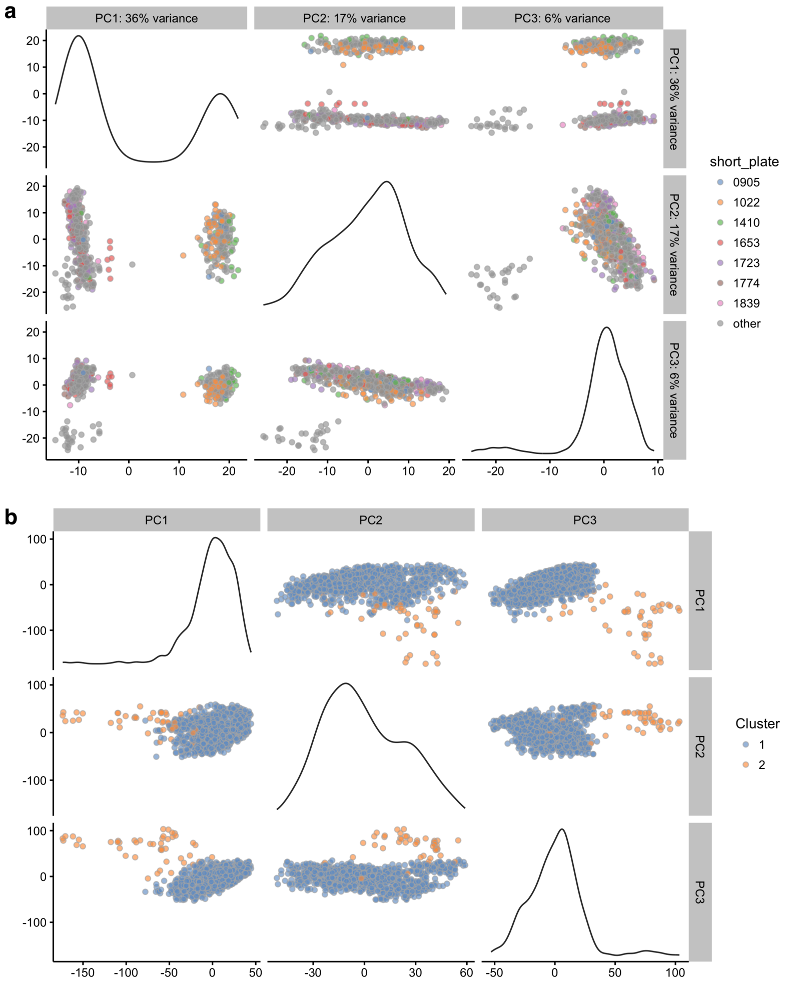

Supplementary Figure 4: Principal Components Analysis of batch effects in TCGA COAD and BRCA data. PCA representations of the COAD (**a**) and BRCA (**b**) datasets, coloured by sequenced plate and GMM cluster assignment respectively.

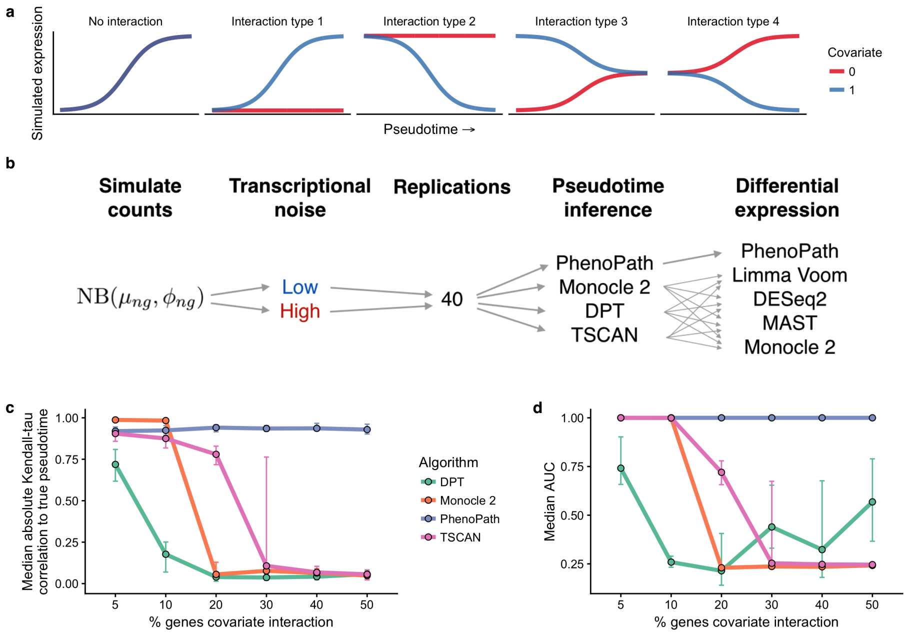

Supplementary Figure 5: Benchmarking PhenoPath performance on simulated data sets. Comprehensive benchmarking of PhenoPath demonstrates the necessity of a joint model of covariates and latent progression. **a** Genes were either simulated as evolving identically along pseudotime regardless of covariate status (*no interaction*) or coming from one of four interacting types. **b** Multiple datasets with differing transcriptional noise were quantified by different pseudotime algorithms and differential expression models leading to 35,520 different workflows. **c** Median Kendall's-τ to true pseudotime for 200 cells in the low noise case. For the smallest fraction of genes exhibiting interactions Monocle 2 performs best, though PhenoPath maintains the best overall performance as the fraction of interactions increases. Error bars show the 95% interval over 40 replications. **d** Area under the curve for detecting interactions shows good performance for Monocle 2, PhenoPath and TSCAN with a low fraction of genes interacting for 200 cells in the low noise case with Limma Voom used for DE. However, as the fraction of interactions increases the performance of the other algorithms quickly decreases, with some performing worse than would be expected at random.

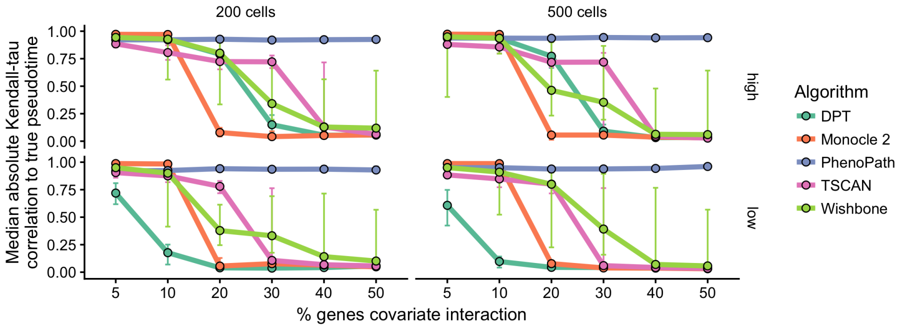

Supplementary Figure 6: Comparison of pseudotime algorithm performance in the presence of interactions. Performance of various pseudotime algorithms as a function of the fraction of genes exhibiting interactions, for both the low and high noise simulation cases and with 200 and 500 cells.

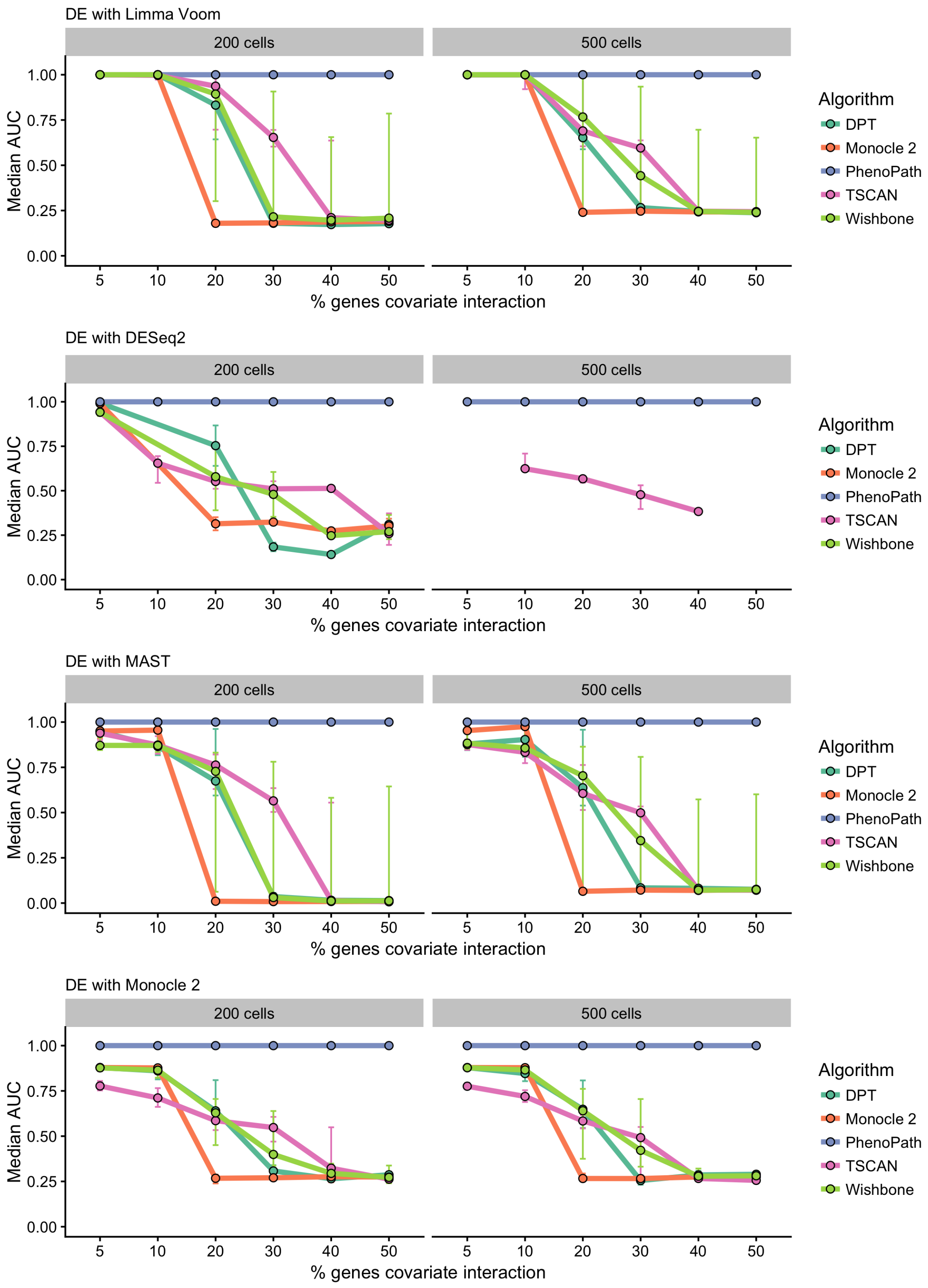

Supplementary Figure 7: Comparison of joint pseudotime and differential expression pipeline performance for detecting the presence of interactions. AUCs for detecting covariate-trajectory interactions for all simulated datasets and pseudotime algorithms with differential expression performed using Limma Voom, DESeq2, MAST, and Monocle 2 for the *high* noise regime. DESeq2 returned `NA` p-values for many fits due to high magnitude outliers driven by the large overdispersion present (TSCAN only returns pseudotimes for a subset of samples which we assume resulted in exclusion of the "unusual" samples).

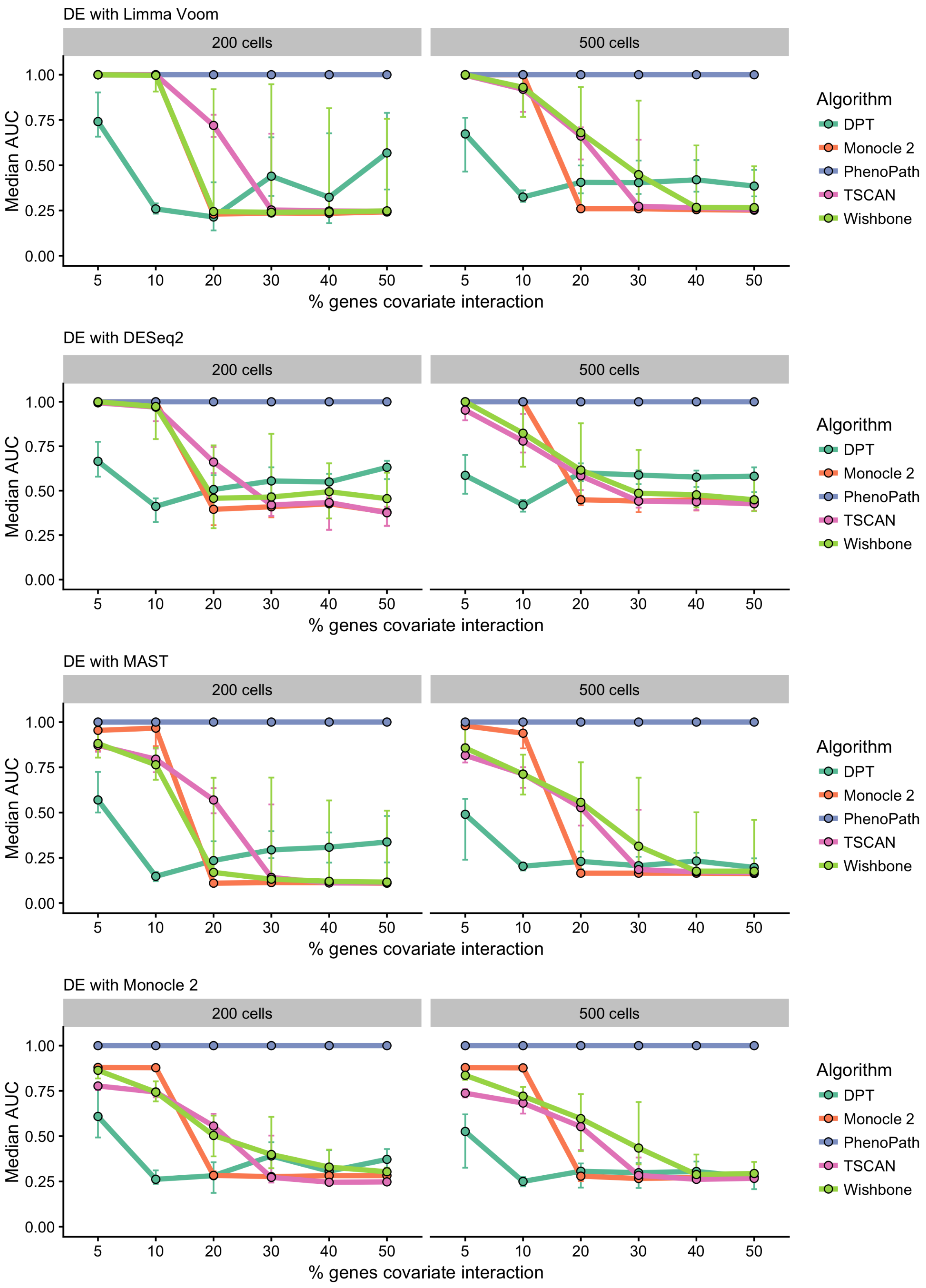

Supplementary Figure 8: Comparison of differential expression analysis algorithm performance for detecting the presence of interactions. AUCs for detecting covariate-trajectory interactions for all simulated datasets and pseudotime algorithms with differential expression performed using Limma Voom, DESeq2, MAST, and Monocle 2 for the *low* noise regime.

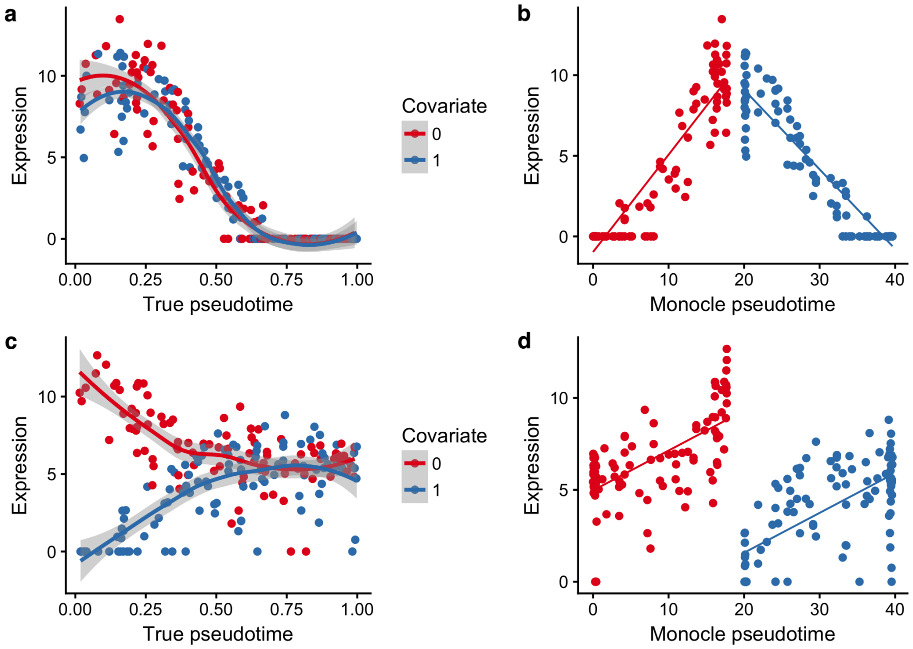

Supplementary Figure 9: Comparison of standard workflow performances in the presence of interactions. A standard pseudotime-DE workflow can lead to severe mis-classification of covariate-trajectory interactions. **a** Simulated expression of a gene that exhibits covariate-independent regulation over pseudotime, as a function of simulated pseudotime. **b** The same gene's expression as a function of the Monocle-inferred pseudotime. **c** Simulated expression of a gene that exhibits covariate-dependent regulation over pseudotime, as a function of simulated pseudotime. **d** The same gene's expression as a function of the Monocle-inferred pseudotime.

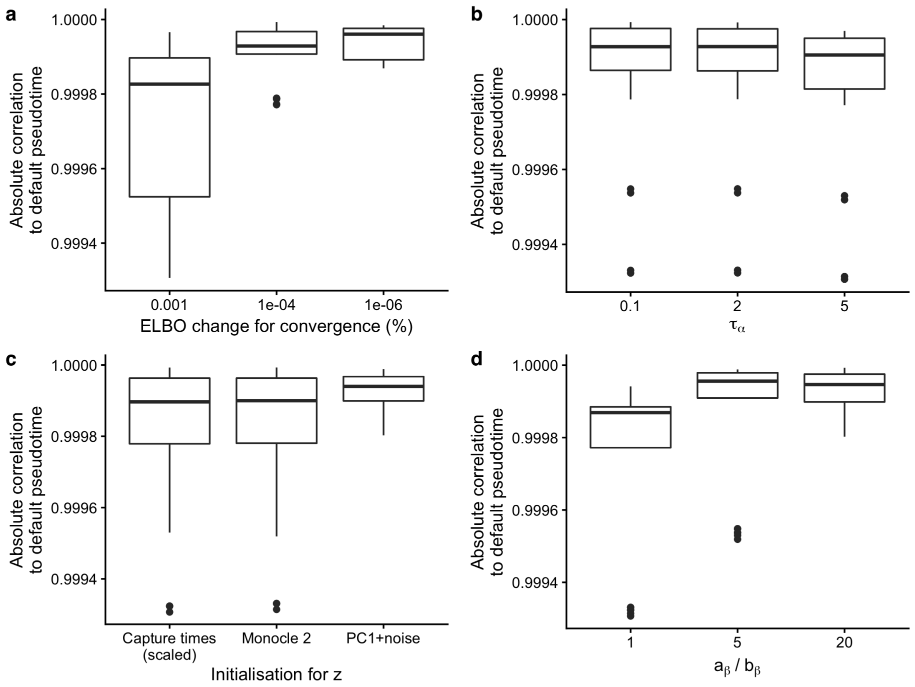

Supplementary Figure 10: Analysis of PhenoPath performance using different initialisations. A total of $3^4 = 81$ different initialisations were used for PhenoPath fitted to the mouse dendritic cells dataset [1], across a range of parameter values for the threshold change in the ELBO to decide convergence (**a**), the hyperparameter τ_α (**b**), the initialisation of the latent space (**c**), and the mean of the Gamma prior distribution on the ARD precisions on β (**d**). Boxplot lower and upper hinges correspond to the first and third quantile. Upper and lower whiskers correspond to 1.5 times the inter-quartile range from the hinge. Data outside this range is considered an outlier.

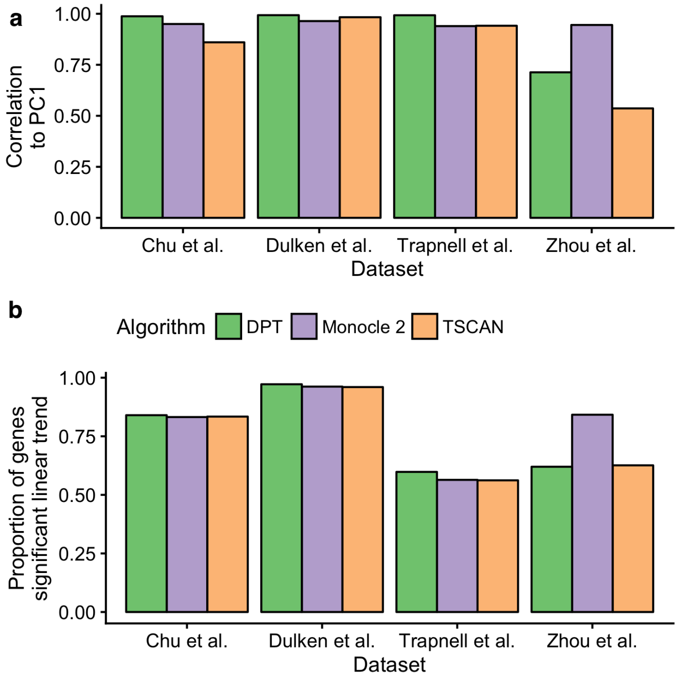

Supplementary Figure 11: Comparison of non-linear pseudotime algorithms with principal components analysis. Single-cell pseudotime is approximately linear. **a** The correlation to the first principal component of the data across the algorithms Monocle 2, DPT, and TSCAN for four single-cell datasets. **b** The proportion of the transcriptome that exhibits a significant linear trend for each pseudotime algorithm and dataset (FDR = 5%).

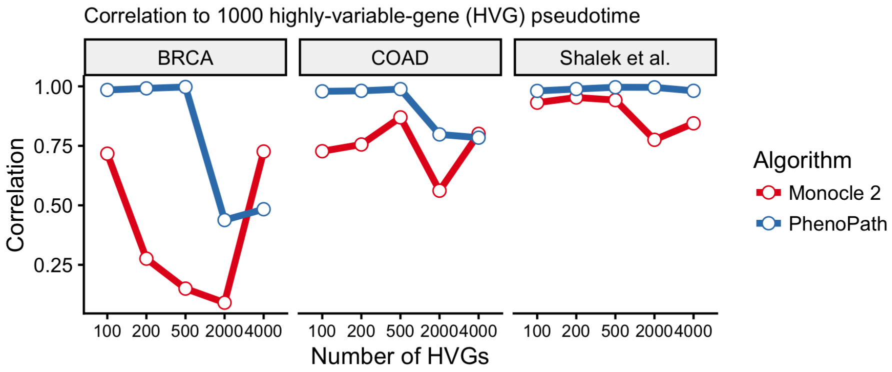

Supplementary Figure 12: Comparison of pseudotime estimation as a function of varying data input. Correlation of the inferred latent space (z) for PhenoPath using a given number of highly variable genes across the three datasets studied to the result using 1000 highly variable genes (blue). In general this is more robust than the same analysis using Monocle 2 (red).

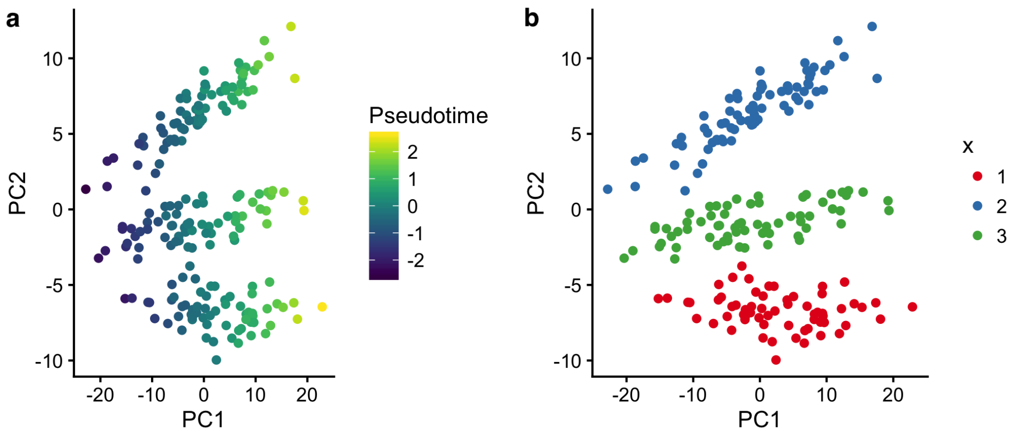

Supplementary Figure 13: Simulated data illustration. PCA plot of the simulated toy dataset, coloured by pseudotime (**a**) and covariate status (**b**).

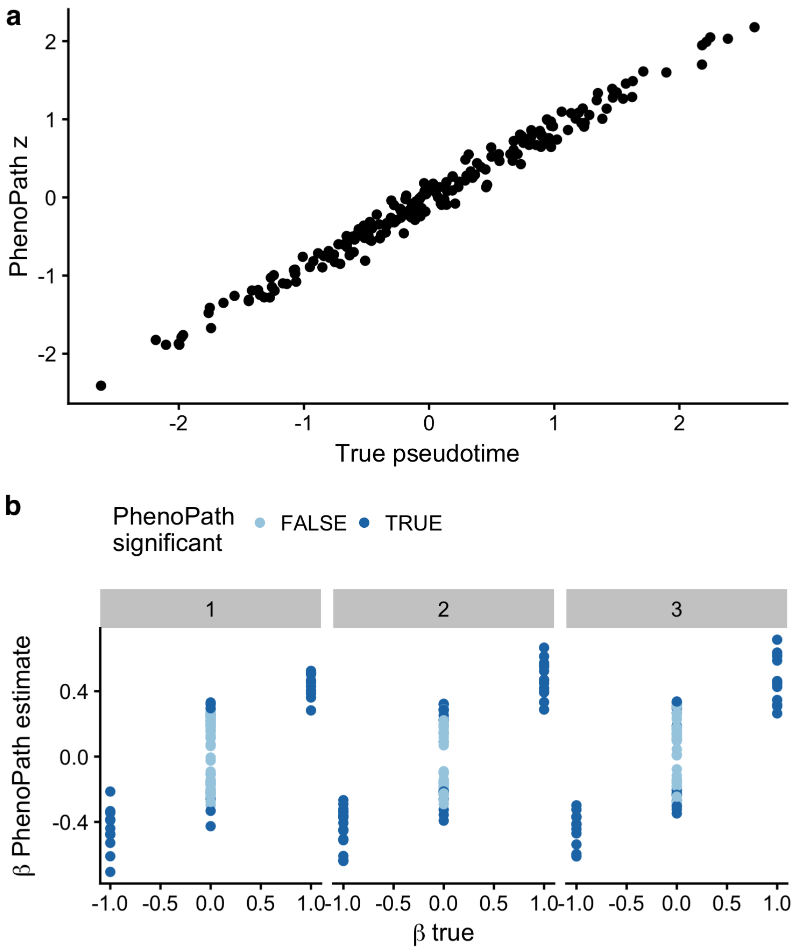

Supplementary Figure 14: PhenoPath analysis of categorical covariate data. **a** The inferred pseudotime against the simulated pseudotime. **b** The inferred β interaction parameters compared to the true values.

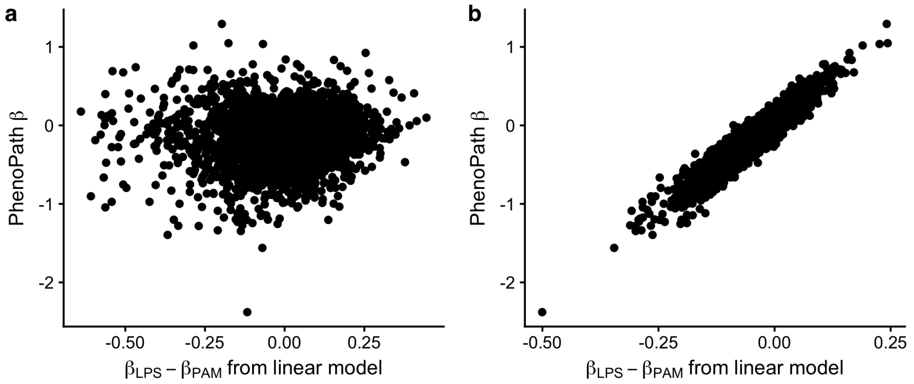

Supplementary Figure 15: Comparison of split-data analysis with PhenoPath integration. **A** Comparison of $\hat{\beta}_{\text{LPS}} - \hat{\beta}_{\text{PAM}}$ values to PhenoPath β interaction coefficients in the intial analysis. **B** The same comparison after "re-orientating" the LPS trajectory.

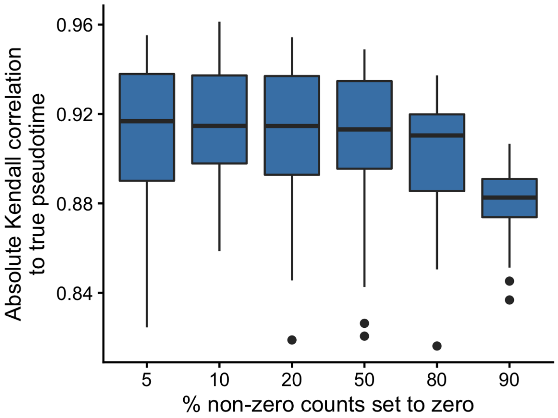

Supplementary Figure 16: Effect of zero-inflation (dropout) on PhenoPath. Boxplots showing correlation to true pseudotime across simulations with different percentage of zero counts. Lower and upper hinges correspond to the first and third quantile. Upper and lower whiskers correspond to 1.5 times the inter-quartile range from the hinge. Data outside this range is considered an outlier. Setting non-zero measurements to zero in simulations does not significantly affect the accuracy of PhenoPath.

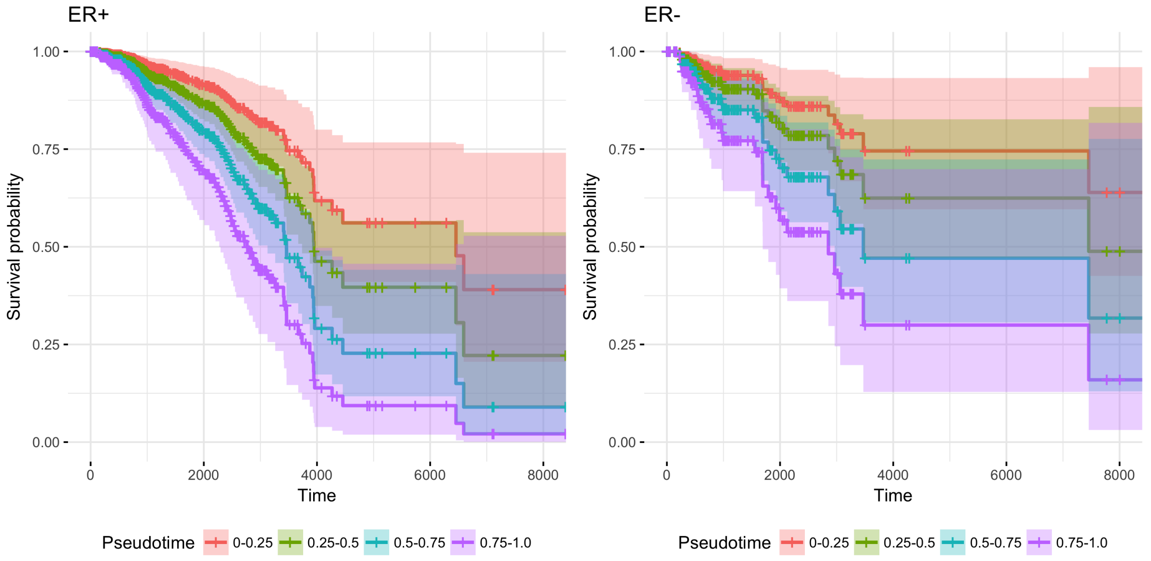

Supplementary Figure 17: TCGA BRCA Survival Analysis. A stratified (by ER status) Cox Proportional Hazards models was fit using PhenoPath derived pseudotimes as a covariate.

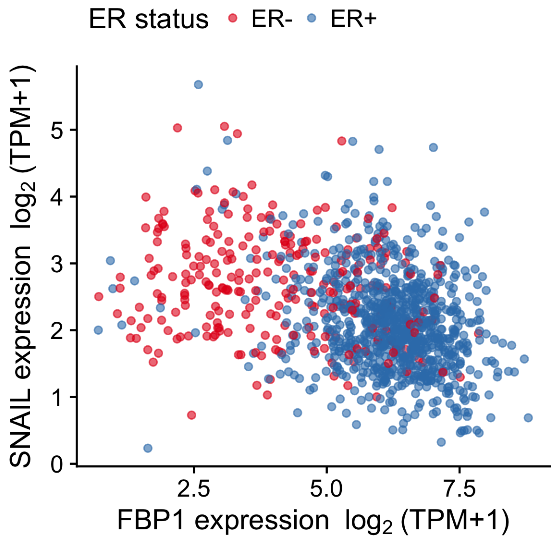

Supplementary Figure 18: ER status marker expression. FBP1 expression is inversely correlated with Snail in ER- breast cancers but shows no dependence in ER+ breast cancers.

List of Tables

1 **PhenoPath hyperparameter specification.** Default hyperparameter values in the PhenoPath model. Note that τ_μ constrained to 0 by default. 40
2 **PhenoPath parameter initialisation.** Default parameter initialisation in the PhenoPath model. 41
3 **Simulation parameters.** Values used for simulation and inference. 42

Hyperparameter	Default value
τ_α	1
τ_λ	1
τ_μ [1]	1
a	2
b	2
a_β	10
b_β	1
q_n	$0 \; \forall n$
τ_q	1

Supplementary Table 1: PhenoPath hyperparameter specification. Default hyperparameter values in the PhenoPath model. Note that τ_μ constrained to 0 by default.

**Parameter (of approx. dist.)**	**Initialisation**
$\mathbf{m}_z$	First principal component of data
$\mathbf{s}_z$	0.1
$\mathbf{a}_\tau$, $\mathbf{b}_\tau$	1
$\mathbf{m}_\alpha$	Coef. from regressing $\mathbf{y} \sim \mathbf{x}$
$\mathbf{s}_\alpha$	0.1
a_χ	0.1
b_χ	0.01
$\mathbf{m}_\beta$	0
$\mathbf{s}_\beta$	0.1
$\mathbf{m}_\lambda$	Coef. from regressing $\mathbf{y} \sim \mathbf{m}_z$
$\mathbf{s}_\lambda$	0.1

Supplementary Table 2: PhenoPath parameter initialisation. Default parameter initialisation in the PhenoPath model.

**Simulation / inference step**	**Values**
% of genes showing covariate-dependent behaviour	5, 10, 20, 30, 40, 50
Noise regime	Low ($\phi = \mu_{ng}/3 + 1$), High ($\phi = 1$)
Number of cells	200, 500
Replicates	40
Pseudotime inference	PhenoPath, Monocle 2, TSCAN, DPT
Differential expression	PhenoPath, DESeq2, Limma Voom, MAST, Monocle 2

Supplementary Table 3: Simulation parameters. Values used for simulation and inference.
